# Deletional Protein Engineering Based on Stable Fold

**DOI:** 10.1371/journal.pone.0051510

**Published:** 2012-12-11

**Authors:** Govindan Raghunathan, Nagasundarapandian Soundrarajan, Sriram Sokalingam, Hyungdon Yun, Sun-Gu Lee

**Affiliations:** 1 Department of Chemical Engineering, Pusan National University, Busan, South Korea; 2 School of Biotechnology, Yeungnam University, Gyeongsan, South Korea; Berlin Institute of Technology, Germany

## Abstract

Diversification of protein sequence-structure space is a major concern in protein engineering. Deletion mutagenesis can generate a protein sequence-structure space different from substitution mutagenesis mediated space, but it has not been widely used in protein engineering compared to substitution mutagenesis, because it causes a relatively huge range of structural perturbations of target proteins which often inactivates the proteins. In this study, we demonstrate that, using green fluorescent protein (GFP) as a model system, the drawback of the deletional protein engineering can be overcome by employing the protein structure with high stability. The systematic dissection of N-terminal, C-terminal and internal sequences of GFPs with two different stabilities showed that GFP with high stability (s-GFP), was more tolerant to the elimination of amino acids compared to a GFP with normal stability (n-GFP). The deletion studies of s-GFP enabled us to achieve three interesting variants viz. s-DL4, s-N14, and s-C225, which could not been obtained from n-GFP. The deletion of 191–196 loop sequences led to the variant s-DL4 that was expressed predominantly as insoluble form but mostly active. The s-N14 and s-C225 are the variants without the amino acid residues involving secondary structures around N- and C-terminals of GFP fold respectively, exhibiting comparable biophysical properties of the n-GFP. Structural analysis of the variants through computational modeling study gave a few structural insights that can explain the spectral properties of the variants. Our study suggests that the protein sequence-structure space of deletion mutants can be more efficiently explored by employing the protein structure with higher stability.

## Introduction

Protein engineering tools have enabled not only the generation of valuable proteins with new functions and stabilities but also the various fundamental studies on protein structure, function, stability and evolution [1], [2]. In general, the efficiency of a protein engineering method relies on the diversity of protein sequence-structure space, because the chance to find out new properties may be increased by exploring more diverse protein sequence-structure space [3]. Substitution mutagenesis is a major tool which has been employed in various protein engineering studies. The approach induces the diversification of target protein sequence-structure space by altering the amino acid residues in natural sequence with other amino acids, which allows for the modulation of target protein function and stability. Indeed, it has tremendously contributed to the development of various academic and industrial fields related to protein engineering and science.

Deletion mutagenesis is another type of mutation used in protein science and engineering. The deletion approach has been mainly employed in mapping the function of a target protein, which significantly contributed to elucidating the relation between protein sequence, structure and function [4]. On the other hand, the deletion mutagenesis has also been recognized as an important protein engineering tool. In particular, deletion mutagenesis is known to induce different structural changes in target proteins from those caused by substitution mutations, which provides important tools for altering protein structures and functions in directions not achievable with substitutions alone [4]. Protein properties such as stability, activity, interface, and size could be changed by the deletion mutagenesis such as N- or C-terminus deletions, loop deletions and random deletions [5], [6], [7], [8], [9], [10], [11]. In addition, protein structural database indicate that deletion mutations are also involved in protein sequence evolution in nature [11], suggesting that deletion mutations can be included in the mutagenesis step of directed evolution. Despite these potentials of deletion mutagenesis in protein sequence engineering and evolution, the approach has not been popularly used compared to substitution mutagenesis method because deletions lead to relatively large structural perturbations, and so the target proteins are prone to be inactivated [12], [13], [14].

Recently, there have been theoretical and experimental demonstrations that protein stability promotes the evolvability of proteins [15], [16], [17], [18]. The concept is that a protein’s capacity to evolve is enhanced by the mutational robustness of stable protein. Although the protein stability-evolvability relationship is not absolutely clear yet, the reports sufficiently provided the insight that the protein engineering and design tasks can be facilitated using stable protein folds with high stability. Indeed, the concept was successfully employed in some typical protein engineering studies based on canonical amino acid mediated substitution mutagenesis [19], [20], [21]. We also demonstrated that the concept could be applied to non-canonical amino acid mediated protein engineering approach [22], which permitted the generation of functional proteins with unnatural properties efficiently [23], [24].

In this study, we show that the protein stability-evolvability concept can also be used to overcome the drawback of the deletion mutagenesis method mentioned above. Our idea is that the use of a more stable fold may allow for more deletions without perturbing the protein’s native structure too much, which may confer us more chances to generate deletion mutants with functional and varied properties. To demonstrate this, green fluorescent proteins (GFP) with different stabilities were mutated by deletion mutations as a model system. We systematically investigated the effect of GFP stability on the tolerance to N-terminal, C-terminal, and internal loop sequence deletions. From the deletion study using a highly stable GFP fold, some interesting variants which cannot be obtained from GFP fold with normal stability could be generated, and their biophysical properties were studied. To understand the properties of generated variants, *in silico* studies on the structural changes of the variants were also performed.

## Materials and Methods

### Materials

The reagents for molecular cloning work were purchased from New England Biolabs (Tokyo, Japan) and chemicals were from Sigma chemicals (St. Louis, MO, USA). pET30b(+) from Novagen was used for the expression of target proteins. The cloning host *Escherichia coli* strain DH5α was used for plasmid DNA preparation in this study. *E. coli* BL21 (DE3) was used as the host for expression of target protein.

### Construction and Expression of s-GFP, n-GFP and Variants

Primers were designed for specific deletions and their sequence details were provided in S**upporting Information S1** along with target genes. The genes of n-GFP variants and s-GFP variants were respectively PCR amplified from pQE80-GFP [25] and pQE-80L-GFPhs1 [22] using the designed primers and cloned into Nde I and Xho I restriction sites of pET30b(+). The internal deletions were made using overlap extension PCR [26]. The constructs had N- or C-terminal hexahistidine tag for affinity purification. The PCR amplified genes were cloned into pET30b(+) and over-expression of s-GFP, n-GFP and deletion variants were carried out in *E. coli* BL21 (DE3). *E. coli* BL21 (DE3) harboring the variants were grown at 37°C and induced at 0.6–0.8 O.D_600_ with 1 mM IPTG for 5 hrs. For SDS-PAGE analysis, 3 O.D_600 nm_ of over expressed cells were collected by centrifuging at a speed of 5000 rpm for 10 min at 4°C and lysed using BugBuster™ Protein Extraction Reagent (Novagen). The lysate was centrifuged at 13,000 rpm for 30 min to separate soluble and insoluble fractions, and analyzed on SDS-PAGE gel. For affinity column purification of protein, cells were grown at 37°C and induced at 0.6–0.8 O.D_600 nm_ for 18–48 hrs at 15°. The cells were collected by centrifugation at 5000 rpm for 10 min at 4°C after overexpression and lysed using French press (Thermo Scientific). The soluble protein fractions were purified by Ni-NTA column chromatography (GE Healthcare Bio-Sciences, Sweden) by standard protocol. Elution fractions were analyzed by SDS-PAGE and dialyzed against phosphate buffered saline (PBS) pH 7.4.

### Measurement of Fluorescence Intensity

The fluorescence of s-GFP, n-GFP and deletion variants were recorded on Perkin Elmer/Wallac Victor 2 Multilabel Counter (1420–011) by measuring the fluorescence intensity by exciting at 485 nm and emission at 515 nm with excitation/emission slits of 5.0 nm. The whole cell fluorescence assay was performed by suspending the cell pellet of 200 µl over expressed culture in PBS for measurement. The excitation/emission spectra of protein were recorded on PerkinElmer LS-55 Fluorescence spectrometer attached with FL WinLab™ software for spectral analysis. For excitation scan, the emission wavelength was set at 560 nm with slit of 2.5 nm except for s-C225 (10 nm). In emission scan, the emission wavelength was scanned by exciting at 440 nm, between 450–600 nm with excitation slit of 2.5 nm except for s-C225 (10 nm). The scan speed for both spectral scanning was 50 nm/min.

### Denaturation and Refolding of GFP Variants

Purified GFP variant proteins (30 µM) each were denatured under harsh condition in PBS (pH 7.4) containing 8 M urea and 5 mM DTT unfolded at 95°C for 5 min. Urea denatured samples were renatured at room temperature by 100 fold dilution into PBS containing 5 mM DTT without urea. The concentrations of denatured proteins were adjusted to 0.3 µM and recovered fluorescence was measured using PerkinElmer LS 55 Fluorescence spectrometer (490 nm excitation, 511 nm emission, 2.5 nm (10 nm for s-C225) excitation/emission slit) for 30 min with an interval of 3 sec. The recovered fluorescence was normalized by dividing with maximum fluorescence value of the spectra. The normalized values were fitted with Sigma Plot (Systat Software Inc., CA) using equations as described by previous report [27].

### Equilibrium Refolding Plot

Column purified proteins at a concentration of 100 µM in 1xPBS (pH 7.4) were diluted 5 fold in 10 M urea dissolved in refolding buffer (25 mM Tris pH 7.5, 0.2 M NaCl, 5% Glycerol, 1 mM DTT) to get final concentration of 8 M urea and unfolded at 95°C for 5 min. Fraction refolded was measured by diluting urea denatured proteins into refolding buffer containing 5 mM DTT to various concentration of urea (1 M–6 M) by allowing to refold for 24 hrs at 15°C. Fluorescence was measured by exciting at 485 nm and emission at 515 nm with excitation/emission slits of 5.0 nm and was recorded on Perkin Elmer/Wallac Victor 2 Multilabel Counter (1420–011). The concentration of urea at which the 50% of initial fluorescence was recovered after 24 hrs incubation is termed C_0.5_. C_0.5_ was determined by sigmoidal fit using sigma plot (Systat Software Inc., CA) [27].

### Fluorescence Microscope Imaging

Liquid culture of s-GFP and its variants of N-terminal and C-terminal deletions were spread with loop on clean glass slides and air dried for 5–10 min. The slides mounted with samples were observed under 40× magnification with 10× objective using Zeiss Axioplan 2 Fluorescence Microscope. The fluorescent cells were captured with digital imaging system and analyzed using Axiovision Rel. 4.8.2, software equipped with microscope.

### Measurement of Circular Dichroism Spectrum

Protein samples were buffer exchanged to 10 mM sodium phosphate (pH 7.4) and the concentration was adjusted to 0.2 mg/ml. CD experiments were performed on JASCO J-715 Spectropolarimeter with a Jasco PTC 348WI temperature controller. The Far-UV spectra (240–200 nm) were measured in 0.1 cm path length quartz cuvette filled with 0.2 mg/ml protein solution. The secondary structure content from the Far-UV spectra was estimated through DichroWeb [28], [29] server with 7 set of reference in Contin program.

### Molecular Modeling of s-GFP and its Variants

The three dimensional structure of the GFP variant, s-GFP was modeled using the template structure of superfolder GFP (PDB ID 2B3P) [27]. The Build mutant protocol from the Protein modeling module of the Discovery Studio 2.0 (Accelrys Inc., San Diego, CA, USA) was used to model the s-GFP structure with the following steps. In the first step, the mutations required to represent the s-GFP were incorporated by replacing those amino acids of the template superfolder GFP structure. Second, the modified structure was optimized using the simulated annealing algorithm only for the mutated residues and its surrounding atoms within the radius of 4.5 Å so that the newly introduced amino acid positioned properly without any steric clash with the surrounding amino acids. Finally, energy minimization was performed for the entire s-GFP structure in order to optimize the overall structure for the introduced mutations. The conditions used for energy minimization were as follows; the atoms were typed with Momany & Rone CHARMm force field, non-bonded interactions cutoff were set to 10 to 12 Å, solvent conditions used were distance-dependent dielectrics with a dielectric constant set to 4 to mimic the screening effects of the solvent at the crude level, energy minimization cycles were carried out until the RMS gradient tolerance was ≤0.1 kcal mol ^−1^Å ^−1^. This modeled s-GFP structure having chromophore was used as the template for modeling the terminal deletion variants of GFP by deleting the corresponding residues mentioned in the Table 1 for each variant. For the modeling of internal deletion variants, the primary sequences after deleting the residues mentioned in the Table 1 for each variant were prepared and modeled using the s-GFP as the template structure with the help of Build homology module of the Discovery Studio 2.0. All the deletion variant structures were energy minimized using the same procedure mentioned above for the energy minimization of s-GFP. The solvent accessible surface area were predicted using the 1.4 Å probe radius, and the amino acid side chains with more than 25% accessibility to this probe radius were considered to be surface exposed residues in this study. The bond distance between N-O for an electrostatic pair to form a strong salt-bridge was considered to be ≤4 Å, and for the hydrogen bond, the distance between the donor and the acceptor atom was considered to be ≤2.5 Å [30], [31].

**Table 1:** Comparison of relative whole cell fluorescence of s-GFP, n-GFP and deletion variants.

Deleted sequences	Name & Relative fluorescence/au (%) of s-GFP variants^[a]^		Name & Relative fluorescence/au (%) of n-GFP variants^[b]^	
No deletion	s-GFP	100	n-GFP	100
2–11	s-N11	80	n-N11	0
2–14	s-N14	35	n-N14	0
2–15	s-N15	0	n-N15	0^[c]^
225–238	s-C225	18	n-C225	0
224–238	s-C224	5	n-C224	0^[c]^
223–238	s-C223	0	n-C223	0^[c]^
76–81	s-DL1	5	n-DL1	-^[+]^
83–88	s-DL2	0	n-DL2	-^[+]^
132–139	s-DL3	2	n-DL3	-^[+]^
191–196	s-DL4	81	n-DL4	0
2–11, 227–238	s-N11C227	36	n-N11C227	0

The relative fluorescence (in arbitrary units) is defined as the whole cell fluorescence compared with fluorescence of cells expressing s-GFP^[a]^ and n-GFP^[b]^ respectively. All the fluorescence values were normalized by the O.D_600 nm_ of expressed cells. ^[c]^Deletion of preceding residues in primary structure of n-GFP resulted in loss of fluorescence and experiments were not carried out. ^[+]^Reported elsewhere [6] that these loop deletions makes the protein non-functional.

### Molecular Dynamic Simulation of s-GFP and s-DL4

The three dimensional structure of s-GFP and s-DL4 were modeled with template crystal structure of GFP (PDB ID 1GFL) [32] which does not contain the chromophore using the same procedure mentioned above for modeling the s-GFP with chromophore. The MD simulation was performed using the GROMACS 4.5.5 with GROMOS96 53a6 as the force field [33]. The protein was solvated using the SPC/E water and the system was neutralized with Na^+^ as counter ions. The system was energy minimized before simulation to avoid bad contacts and inappropriate geometry using the steepest descent algorithm for 2000 steps. Position restrained simulation was performed for equilibrating the system using the *NVT* and *NPT* ensemble, each for 100 ps. Unrestrained simulation was performed for 10 ns with Berendsen thermostat of 300 K and the pressure was maintained at 1.0 bar with Parrinello-Rahman pressure coupling factor. The trjconv, g_rms, and g_rmsf utilities of GROMACS 4.5.5 were used to analyze the MD results.

## Results

### GFP Folds Used in this Study

Two different GFP sequences, i.e. GFP_mut3.1b_ and GFPhs1, with different stabilities were employed to compare the effect of tolerance to deletion mutation. GFP_mut3.1b_, a S65G/S72A mutant of wild type GFP, is one of the rapid folding GFPs engineered by directed evolution [34] and has been used in various studies [35], [36]. GFPhs1 is a derivative of superfolder GFP constructed by introducing 11 superfolder GFP mutations [27] and other 2 mutations known to stabilize GFP structure into GFP_mut3.1b_ [22]. The GFPhs1 exhibited higher stability and mutational robustness compared to GFP_mut3.1b_, and their detailed sequences and biophysical properties were described in our previous report [22]. In this study, the GFP_mut3.1b_ and GFPhs1 were used as a GFP fold with normal stability (n-GFP) and a GFP fold with higher stability (s-GFP), respectively.

### Tolerance of s-GFP and n-GFP for Deletion

**Figure 1:**
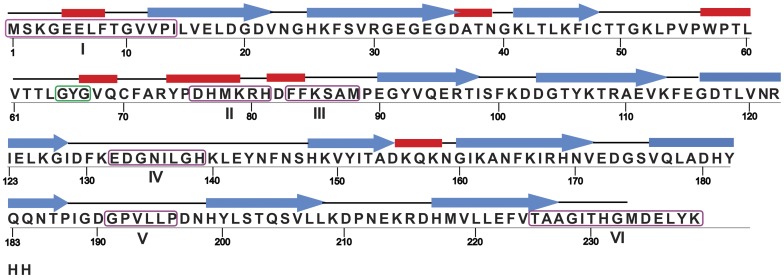
The primary structure and secondary structure map of GFP [**27**] **.** Single-letter amino acid codes are used for representing the primary structure. Green color boxed residues ‘GYG’ represents the chromophore of GFP. Strands are represented in blue arrowhead and helices are indicated in red. The connecting black lines represent the turns and loops which conjoins the adjacent strands. The sequence of s-N14 is devoid of residues in box I. For s-C225, the residues in box VI are deleted. Residues in boxes II, III, IV and V are deleted in s-DL1, s-DL2, s-DL3 and s-DL4 respectively.

**Figure 1** shows the representative secondary structure of GFP, a single chain polypeptide of 238 amino acids with 7 α-helices, 11 β-strands and some connecting loop regions [27], and the primary sequence of s-GFP. The residues at position 65–67 in GFP form fluorophore on spontaneous oxidation and this characteristic spectral signal directly marks the structure and activity of the protein [37]. First, we generated various N-terminal, C-terminal, and internal sequence deletion variants of s-GFP using designed primers, expressed in *E. coli*, and measured their whole cell fluorescence. Based on the results, we constructed corresponding n-GFP deletion variants, and conducted same experiments.

**Table 1** presents the representative results comparing the relative whole cell fluorescence of s-GFP variants with those of n-GFP variants. The dissection of the N-terminal deletions of s-GFP allowed us to identify that the deletion of N-terminus up to 14^th^ amino acid retains the fluorescent activity of s-GFP despite the decrease of fluorescence and the deletion of 2–15 amino acids completely abolished the activity. C-terminal deletion studies revealed that the C-terminal can be eliminated up to the 225^th^ amino acid with fluorescence activity. On the other hand, the deletion variants of 2–11, 2–14 and 225–238 for n-GFP lost their activities completely. We didn’t further dissect the N- and C- terminal deletions for n-GFP, but the results clearly indicate that the higher the protein stability is, the more we can delete the terminals of target proteins.

For the study on the elimination of internal sequences, we deleted four internal sequences (76–81, 83–88, 132–139, 191–196) of s-GFP which generated the mutants of s-DL1, s-DL2, s-DL3, and s-DL4. The 76–81 and 83–88 residues are the sequences that form small α-helices and the 132–139 and 191–196 are the sequences involving large loops of GFP. The sequences are known to be essential in the formation of proper structure of GFP and the deletions of these sequences led to the complete inactivation of GFP [6]. As shown in **Table 1**, among the s-GFP variants individually devoid of four internal loops, s-DL1, s-DL2 and s-DL3 were completely inactivated, but the deletion mutant of 191–196 residues (s-DL4) exhibited about 81% of fluorescent activity of s-GFP. On the other hand, the 191–196 deletion mutant of n-GFP (n-DL4) was completely inactivated. The combinatorial deletions of the internal loop (191–196), N-terminal (2–11 or 2–14), and C-terminal (227–238) for s-GFP were also carried out. Among the variants, only the mutant of 2–11 and 227–238 deletions (s-N11C227) showed about 36% fluorescence activity of s-GFP, and other mutants such as s-N11C227DL4 and s-N14C227 showed 4% and 7% of fluorescence activity respectively. s-N14C227DL4 showed almost complete inactivation (**Figure S1**). On other hand, the deletion mutation of 2–11 and 227–238 for n-GFP (n-N11C227) showed no fluorescence. All these results also clearly suggest that s-GFP is more tolerant than n-GFP against various deletion mutations.

### Expression Analysis of the Deletion Variants

**Figure 2:**
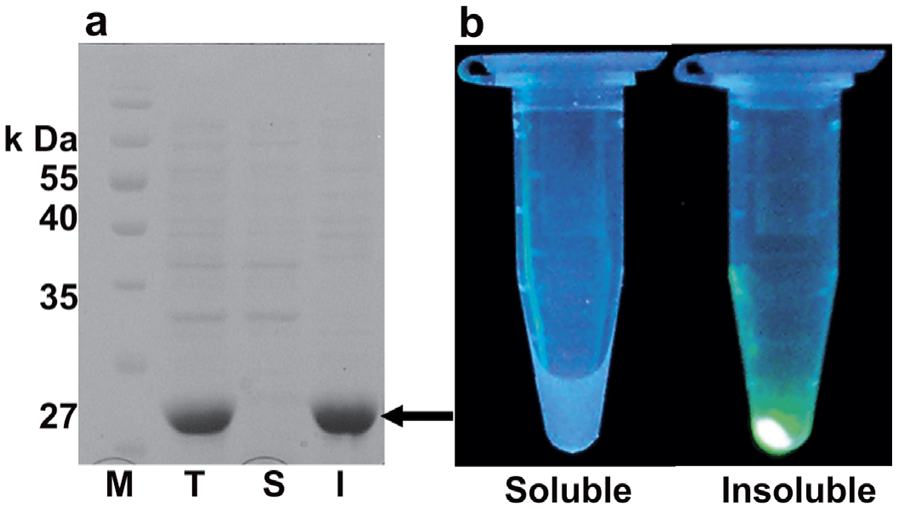
Expression and fluorescence activity of s-DL4. (a) Expression profiles of *Escherichia coli* BL21 (DE3) that produces s-DL4 variant which is devoid of internal loop (191–196). Lane M-Marker, Lane T-total cell protein, Lane S-Soluble fraction, Lane I-Insoluble Fraction. The expressed protein is indicated by arrow (b) UV irradiation of soluble and insoluble fraction of lysed s-DL4 variant to show the activity of the protein.

The SDS-PAGE analyses of the soluble and insoluble fractions for the above mutants were performed and the results are shown in **Figure S2**. In the analyses, we could observe some interesting points. First, most non-fluorescent variants of s-GFP and n-GFP showed insoluble expression of the target protein, but the inactive s-DL1 and s-DL2 mutants showed almost the same soluble expression level of s-GFP (**Fig. S2, (a)**). It is presumed that the deletions of 76–81 or 83–88 for s-GFP didn’t significantly perturb the global structure of s-GFP fold despite the slight structural change that can inactivate fluorescence. Second, the N- or C- terminal deletion variants of s-GFP, i.e. s-N11, s-N14, s-C225, and s-C224, showing decreased fluorescence compared to s-GFP (**Table 1** **)** showed similar soluble expression levels to s-GFP (**Fig. S2, (b) and (c)**). These results suggest that the decreased fluorescent levels of the mutants were not from the decreased soluble expression levels, but from the decreased spectral intensities of the variants. Third, s-DL4, the 191–196 deletion mutant of s-GFP, with an 81% fluorescent level of s-GFP, showed a very intriguing result that the most expressed GFP was detected in insoluble fractions (**Figure 2a**). This suggests that the s-DL4 variant is expressed as insoluble form but functionally active, which was confirmed by detecting the fluorescence predominantly in the insoluble fraction (**Figure 2b** **).**

### Biophysical Properties of s-N14 and s-C225

Among the variants generated in above studies, the two deletion variants of s-GFP, s-N14 composed of 225aa and s-C225 composed of 224aa, were interesting because, as suggested in the previous reports [5], [6], the N-terminal or C-terminal deletion mutants of GFP with reduced size can efficiently be utilized in some biochemical studies. To examine the possibility that s-N14 and s-C225 can be employed in biochemical studies, the biophysical properties of the two mutants were compared with those of n-GFP, one of the GFP variants which have been used in various studies as a folding reporter [35], [36]. Specifically, we investigated the whole cell fluorescence, specific fluorescent activities, fluorescent spectral properties, refolding rates and tolerance to denaturant during refolding of the mutants. s-GFP was also characterized as a control. **Table 2** summarizes the biophysical properties of the selected mutants and the brief description of the results is followed.

**Table 2:** Comparison of biophysical properties of s-GFP, n-GFP and variants.

Property	s-GFP	s-N14	s-C225	n-GFP
Relative whole cell fluorescence/au (%)[a]	100	35	18	24
Specific activity (%)[b]	100	70	22	45
λ_ex/em_ [c]	490/511	490/511	467/506	501/511
Refolding rate×10^−2^s^−1[d]^	2.0	1.8	2.0	0.8
C_0.5_(M)[e]	3.6	2.3	2.1	1.9

[a] The relative fluorescence (in arbitrary units) is defined as the whole cell fluorescence compared with fluorescence of cells expressing s-GFP. All the fluorescence values were normalized by the O.D_600 nm_ of expressed cells.

[b] Specific activity (in arbitrary units/µM of purified protein) is the fluorescence of purified protein compared with the fluorescence of purified protein of s-GFP.

[c] The excitation and emission maxima in units of nanometers.

[d] The refolding rate constant for the fast phase calculated by fitting refolding curve in sigma plot.

[e] Concentration of urea (M) at which 50% of the initial fluorescence is recovered during refolding of urea denatured protein.

**Figure 3:**
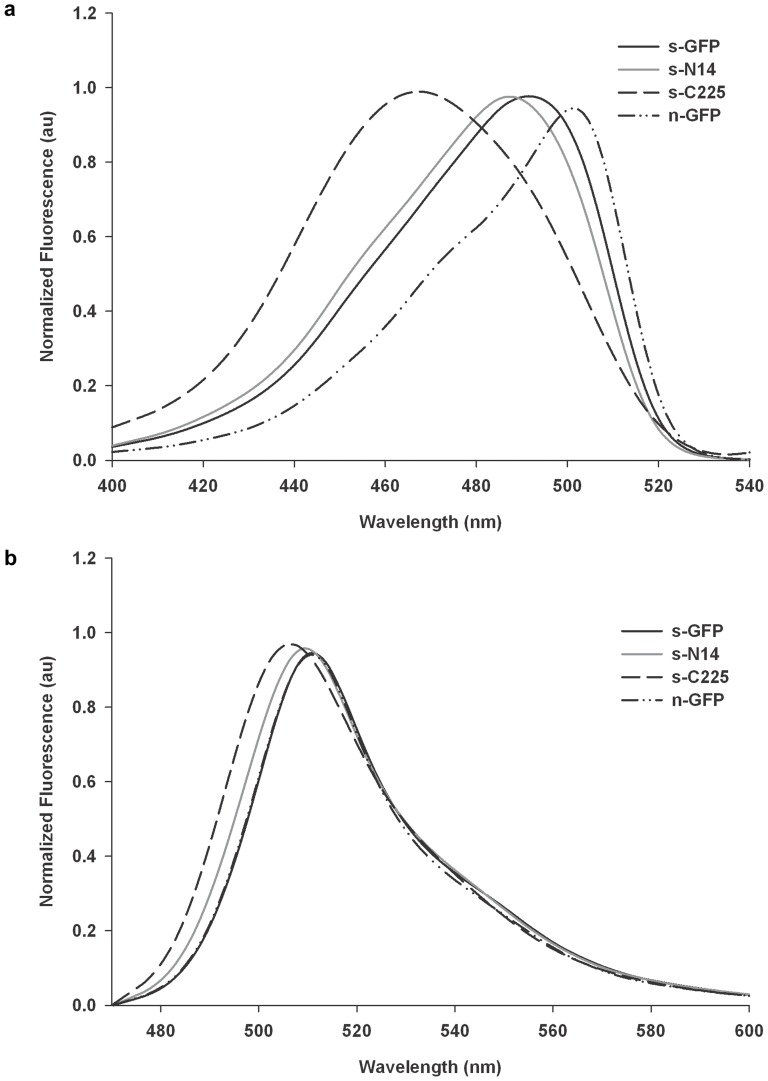
Fluorescence excitation and emission spectra. a) Excitation and b) Emission spectra of s-GFP, s-N14, s-C225 and n-GFP, measured using fluorescence spectrometer. All amplitudes were arbitrarily normalized to a maximum value of 1.0 to show the difference in spectral wavelength not the spectral intensity.

**Figure 4:**
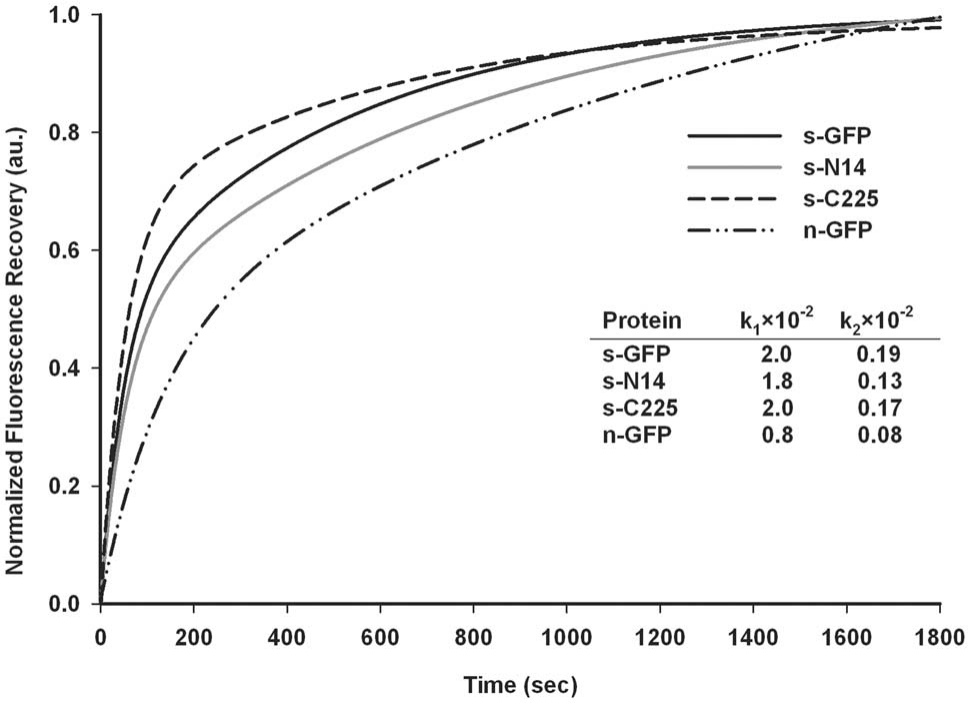
Representative plot of refolding kinetics for s-GFP, s-N14, s-C225 and n-GFP. Refolding kinetics was measured after denaturation in urea (8 M) followed by renaturation by dilution. Normalized fluorescence in arbitrary units (au) was plotted against time. Insert table showing the fast phase and slow phase rates of refolding process.

**Figure 5:**
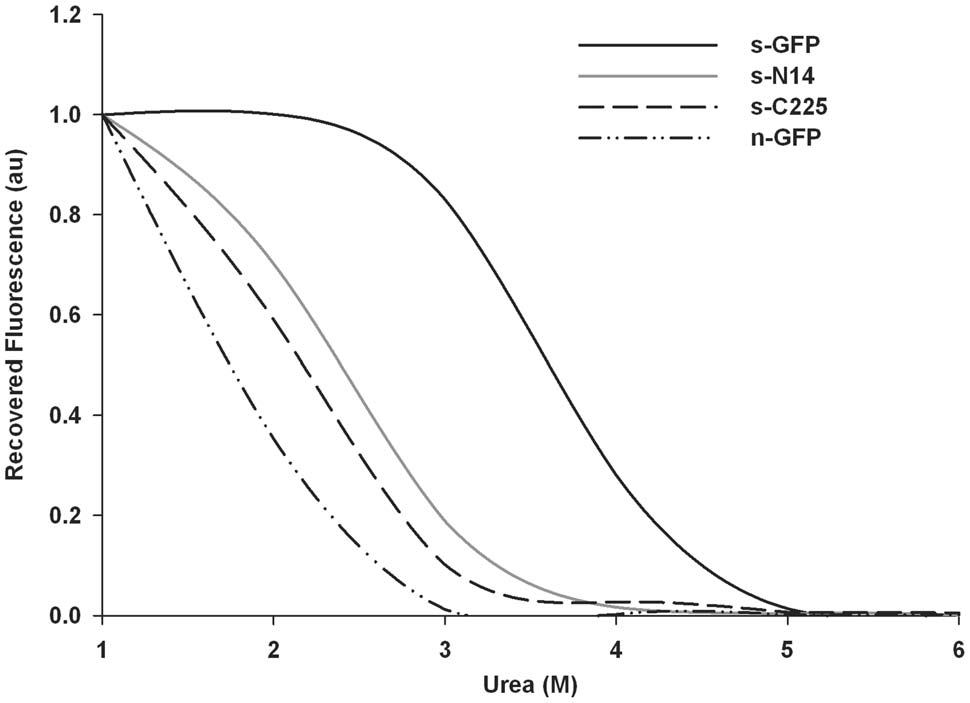
Equilibrium refolding plot for s-GFP, n-GFP, s-N14 and s-C225. Protein samples were denatured in 8 M urea and diluted to different concentration of urea in refolding buffer. Recovered fluorescence normalized by dividing by fluorescence of corresponding non-denatured samples diluted in similar fashion.

The whole cell fluorescence activities of s-N14 and s-C225 were approximately 35% and 18% of s-GFP, but slightly higher and comparable to that of n-GFP, respectively. The specific activities of the purified s-N14 and s-C225 were about 70% and 22% of s-GFP, which corresponded to approximately 1.5 fold higher and two fold lower values of n-GFP, respectively. Here, the lower specific activities of s-N14 and s-C225 than s-GFP support the decreased whole cell fluorescence activities of the mutants despite the comparable soluble expressions described above. When the excitation and emission wavelengths were scanned, the s-N14 mutant showed almost the same properties as that of s-GFP, but the s-C225 exhibited the slight shift in wavelength of the ex/em spectra (**Figure 3**). An *in vitro* refolding study showed that the refolding rates of the two deletion mutants were almost the same as s-GFP and higher than n-GFP **(** **Figure 4**). This result is correlated with the SDS-PAGE analysis almost showing the similar soluble expression levels of the s-GFP, s-N14 and s-C225 described above. In order to test the tolerance of variants to urea during refolding, we performed the experiment of renaturation equilibrium plot against different concentrations of urea and measured the C_0.5′_s of the variants (**Figure 5**). The refolding stability of s-N14 and s-C225 were superior to n-GFP but exhibited reduced stability than s-GFP.

**Figure 6:**
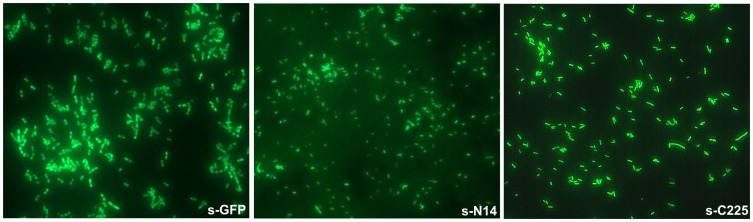
Fluorescence microscope image of s-GFP and deletion variants. s-GFP, s-N14 and s-C225 were employed in live cell imaging by expressing in *E. coli*. The images were captured using fluorescence microscope equipped with digital image analyzer.

Taken together, the biophysical properties of s-N14 and s-C225 were worse than s-GFP, but better or at least comparable to those of n-GFP. These results suggest that the variants generated by the truncation of terminal sequences can be employed in biochemical studies. To confirm the possibility of employment of the deletion variants in biochemical studies, we expressed the variants in *E. coli* and captured the images using fluorescence microscope. It was observed that the fluorescence levels of the deletion mutants were sufficient to perform *in vivo* imaging studies (**Figure** **6**).

### Study on the Structural Perturbations of s-N14 and s-C225

**Figure 7:**
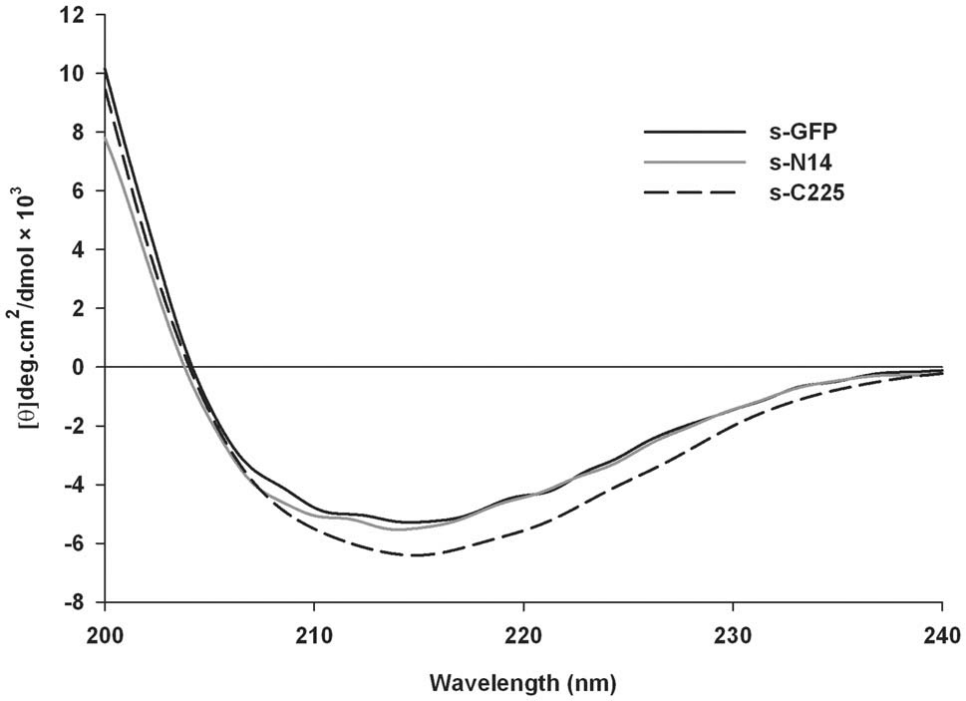
Structural validation by Circular Dichroism spectroscopy. CD spectral analysis of s-GFP, s-N14 and s-C225. Comparison of Far-UV (200–240 nm) CD spectra of s-GFP, s-N14 and s-C225 showed the major peak for secondary structure of β-sheet.

To investigate whether or not there were any significant structural changes occurred due to the deletion of N-terminal and C-terminal of s-GFP, we performed CD spectral analyses of s-GFP, s-N14, and s-C225 (**Figure 7**). Although the analyses of the secondary structure content of the variants from the CD spectral data showed a slight variation of secondary structure amounts which may be caused by N- or C- terminal deletions (**Table S1**), we could not observe prominent structural changes between the s-GFP and the variants. These results indicate that the main β-barrel structure of s-GFP required for the fluorescent activity was almost maintained despite the elimination of the fractions of secondary structures in terminals.

### Structural Analysis of s-GFP and Deleted Variants by Molecular Modeling

As shown in the **Table 1** and **Figure S2**, the N- or C-terminal deletion mutants, i.e. s-N11, s-N14, s-C225, s-C224, s-N11C227, showed similar soluble expression levels but reduced whole cell fluorescence activities compared to s-GFP. In the case of loop deletion mutants, s-DL1 and s-DL2, significant fluorescent activities were not detected despite the soluble expression of the variants. Especially, s-C225 exhibited the shift in em/ex wavelength (**Figure 3**). These results suggest the possibility that the variants folded and formed proper β-barrel structure as demonstrated for s-N14 and s-C225 in the **Figure 7**, but their fluorescent properties were substantially changed. In general, the spectral properties of GFP are known to be sensitively influenced by the arrangement of amino acids and their interactions such as hydrogen bonding and ion pair around chromophore [32], [37], [38], [39]. Therefore, to understand the reduced or lost fluorescence activities of the variants, three dimensional structures of the deletion variants (s-N11, s-N14, s-C225, s-C224, s-N11C227, s-DL1 and s-DL2) were predicted by *in silico* modeling and their structural differences from s-GFP were analyzed. Superfolder GFP (2B3P) was used as the template structure to model the s-GFP because s-GFP is the variant that includes mostly superfolder GFP mutations. Detailed procedures for the structure modeling are described in *Materials and Methods* section. The root mean square deviations of the modeled structures of s-GFP and its variants were found to be less than 1Å compared to their corresponding template.

**Figure 8:**
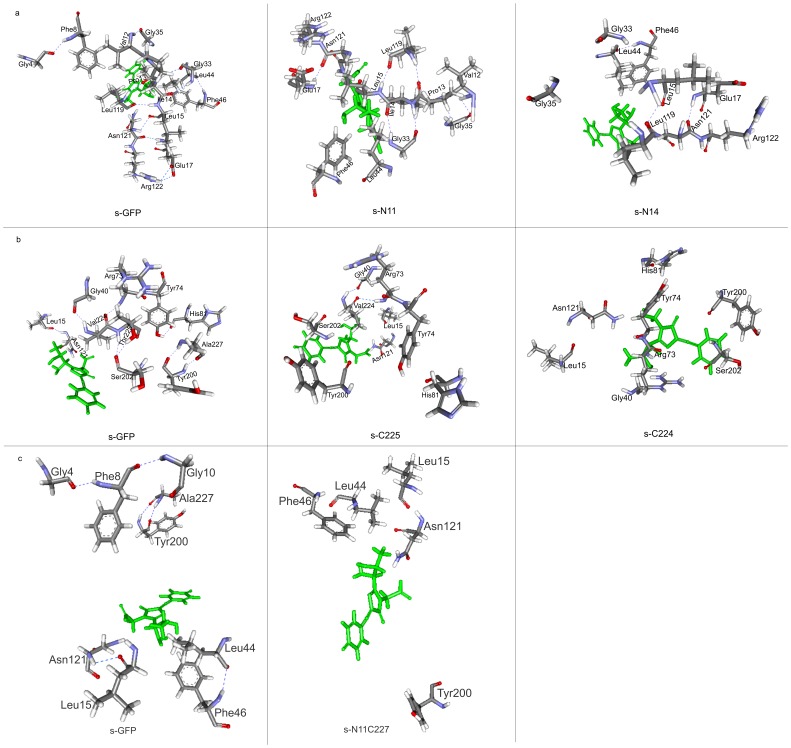
Computational analysis of changes in structural interactions of s-GFP variants. Comparison of lost hydrogen bonding network between s-GFP and deleted variants a) In s-N11 variant, deletion of F8 disrupted the hydrogen bond interaction with G4 along with loss of other interactions as compared with s-GFP. In s-N14 mutants, deletion of V12, P13 and I14 were shown clearly by comparing with s-GFP which resulted in loss of hydrogen bond interaction with G35, L119 and G33 respectivley. b) The interaction map of s-C225 showed the deleted residues T225 and A227, and the lost hydrogen bonds. Deletion of V224, T225 and A227 were shown in the interaction map of s-C224. c) The combined deletion variant s-N11C227 interaction map shows the deleted residues Phe8, Gly4, Gly10 and Ala227 and the interactions they were involved.

The lost and new electrostatic interactions of the N-terminal deletion variants (s-N11 and s-N14) were shown in **Table S2**. The modeled structure of s-N11 has one new hydrogen bond, but it lost 2 salt bridges and 12 hydrogen bonds. About 5 residues of the lost interactions were located within 10Å distance of chromophore. The modeled structure of s-N14 had 2 putative hydrogen bonds besides the loss of original interactions of s-GFP which includes 12 hydrogen bonds and 2 salt bridges. Among the 24 residues involved in the lost hydrogen bonds, 10 residues were found in the 10Å distance of chromophore, and I47 which forms putative hydrogen bonding with T49 was also located within 10Å distance. The interaction map of s-GFP, s-N11 and s-N14 showed that the Phe8 residue and F8–G4 interaction was lost due to the deletion in s-N11, and Phe8, Val12, Pro13, Ile14 residues and their related interactions were lost due to the deletion in s-N14 (**Figure 8(a)**).

The interactions of C-terminal deletion variants (s-C225 and s-C224) were also analyzed using the modeled structures and the changes in interactions were shown in **Table S3**. The energy minimized model of s-C225 was found to lose 9 parent hydrogen bonds and to have one putative salt bridge and 4 putative hydrogen bonds. Among the residues involved in the lost hydrogen bonds, newly generated hydrogen bonds, and newly generated salt-bridges, 9 residues, 5 residues, and 2 residues were found around the chromophore in 10Å distance, respectively. The modeled structure of s-C224 had 3 putative hydrogen bonds and 2 new salt bridges besides the loss of 8 original interactions. When the interactions within the 10Å area of chromophore of s-GFP, s-C225 and s-C224 were compared (**Figure 8(b)**), in addition to the lost and altered hydrogen bond network mentioned in **Table S3**, two residues Thr225 and Ala227 were eliminated in the interaction map of s-C225, and three residues Val224, Thr225 and Ala227 were deleted in s-C224.

**Table S4** shows the lost and new interactions of combined deletion variant s-N11C227. The s-N11C227 was found to lose 14 parent hydrogen bonds and 2 parent salt bridges besides 8 new interactions, which included 6 hydrogen bonds and 2 salt bridges. Among the residues involved in lost interactions, 6 of lost hydrogen bonds, 4 of newly generated hydrogen bonds and 4 of new salt bridges were located within 10Å distance of chromophore. In s-N11C227, the interactions of residues within 10Å distance of the chromophore were mapped and compared with s-GFP, highlighting the elimination of Gly4, Phe8, Gly10 and Ala227 (**Figure 8(c**)).

**Figure 9:**
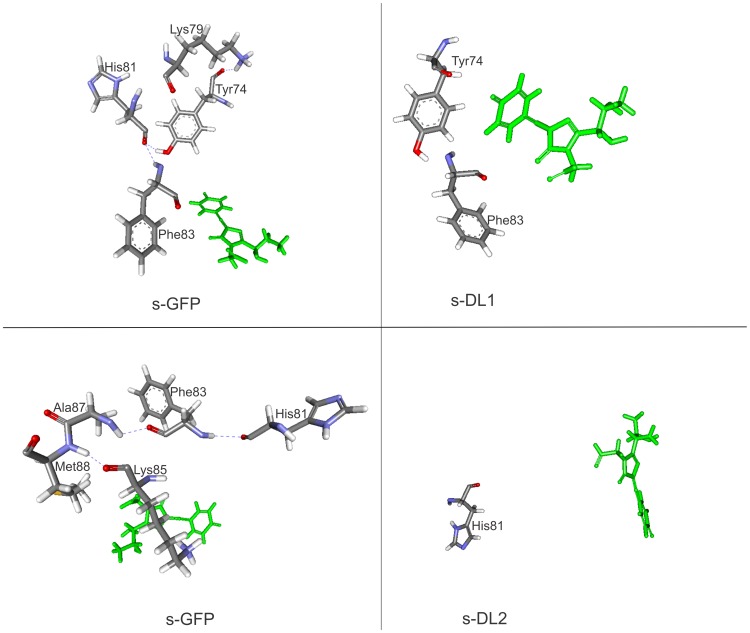
Structural interactions of modeled structure of loop deleted variants. a) In s-DL1, the loop deletion eliminated hydrogen bonds involved by the residues K79 and H81. b) The residues F83, K85, A87 and M88 were deleted in the loop sequence of s-DL2.

**Table S5** shows the lost interactions of loop deleted variants (s-DL1 and s-DL2), and **Figure 9** presents their interaction maps around chromophore. The analysis informed that the residues of the deleted loops resulted in the loss of some amino acid residues and their related interactions around chromophore. In s-DL1, the residues Lys79 and His81 were missing along with their interactions with Tyr74 and Phe83 due to the loop deletion within 10Å distance of chromophore. In s-DL2, the missing of two amino acids (Phe83 and Met88) and loss of 3 hydrogen bonds were found within the 10Å distance of chromophore due to the deletion.

Above molecular modeling analysis gave following probable clues for the changed spectral properties of the variants. First, the structural analysis revealed that some hydrogen bonds, salt bridges and amino acid residues of the s-GFP were lost and reoriented due to the deletions. The loss of electrostatic interactions as well as some amino acid residues was also observed around chromophore in the deletion variants (**Figure 8** **and** **9**). As mentioned above, the chromophore of fluorescent proteins exhibits different fluorescent characteristics depending on the microenvironment around the fluorophore [32], [37], [38], [39]. It is presumed that the deletions changed the packing interactions around the fluorophore environment and led to the reduced fluorescence of the variants. Second, above analysis informed us some correlations between spectral properties of variants and their structural changes around chromophore. An observed trend was that the variants showing almost complete loss of fluorescence lost more amino acid residues and electrostatic interactions around chromophore compared to the variants showing reduced fluorescence. In particular, deletion of C-terminal residues close to the catalytic residue E222 [40] would have affected the electrostatic microenvironment around chromophore more significantly, which might contribute to the shift of λ_em/ex_ wavelengths in addition to the reduced fluorescent activity of s-C225.

### *In silico* Analysis of the Structure and Stability of s-DL4

An interesting result in our study was that the s-DL4 obtained from the internal loop deletion showed active but insoluble production (**Figure 2**). In general, the expression of GFP in *E. coli* leads to the soluble active expression and insoluble inactive expression. Although the insoluble fraction can include active GFP, the ratio is known to be very low [41]. On the other hand, in the case of s-DL4, approximately more than 95% of the expressed insoluble GFP was active when estimated based on the whole cell fluorescence and the amount of expressed protein. Although further detailed studies are needed, it is presumed that the protein sequences for s-DL4 folds into the proper structure of GFP for fluorescence, but the folded structure is prone to be aggregated or multimerized.

**Figure 10:**
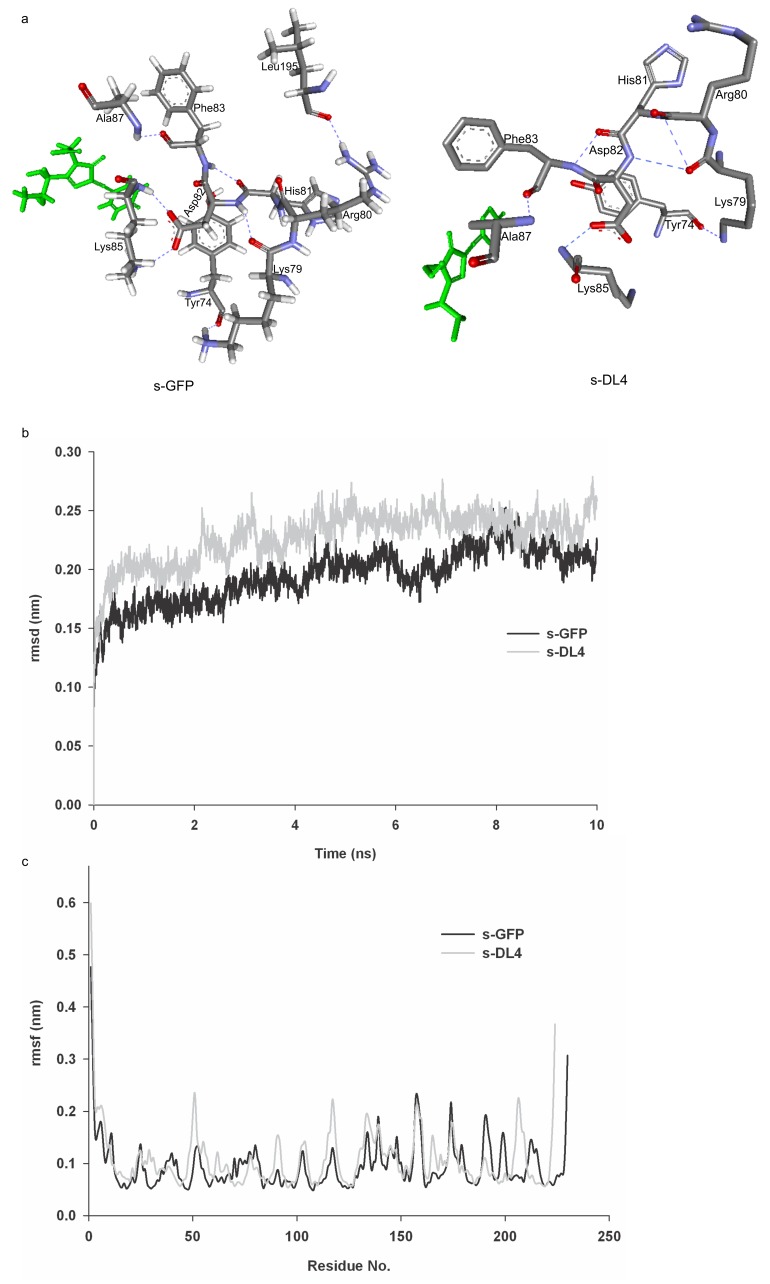
Computational modeling and molecular dynamic simulation of s-DL4. a) Lost hydrogen bonding between R80–L195 is shown in the interaction map with additional interaction surrounding the chromophore. Comparison of b) root mean square deviation (rmsd) and c) root mean square fluctuation (rmsf) of the main chain atoms of s-GFP and s-DL4.

To understand the fluorescence activity of s-DL4 showing almost same activity with s-GFP, the structure of s-DL4 was modeled likewise the other variants. The modeled s-DL4 structure showed that only one hydrogen bond interaction was lost due to deletion of internal loop (191–196). Residue Leu195 had hydrogen bond interaction with Arg80, which was lost due to loop deletion **(** **Figure 10(a)** **)**. The loss of only one hydrogen bond in the deleted sequences for s-DL4 might be the plausible explanation for retaining its fluorescence activity with less structural perturbation. In addition, the lost L195 and related hydrogen bond are not within 10Å distance of chromophore, which also supports the non-changed fluorescent activity of s-DL4.

To find the possible explanation for the aggregation behavior of s-DL4, the structure of s-DL4 was compared with the structure of s-GFP, and structural changes were examined. In this analysis, we focused on examining the structural changes of the exposed hydrophobic amino acids in GFP surface and the amino acids in the GFP dimer interface [32]. Any major structural differences between s-GFP and s-DL4 were not observed in the hydrophobic residues on surface and the residues in dimer interface, which might be caused by the current limitations in the computational structure modeling. To probe the structural perturbations of s-DL4 induced by the loop deletion further, molecular dynamic (MD) simulations study was performed. MD simulations of s-GFP and s-DL4 showed that the main chain of s-DL4 was more dynamically fluctuated than s-GFP (**Figure 10(b)**), which suggests that the loop deletion may destabilize the structure of the folded s-GFP. The analysis of the time-average fluctuations of the individual residues suggested that there were some specific regions showing high fluctuations in the primary sequence of s-DL4 (**Figure 10(c)**). In particular, it was identified that 30 residues of s-DL4 showed fluctuations with the difference of more than 0.5 Å compared to the corresponding residues of s-GFP (**Table S6).** In addition, 9 exposed residues which were reported to be in the dimer interface of GFP [32] and 17 other exposed hydrophobic amino acids of s-DL4 were showing higher fluctuations than s-GFP (**Table S6).** These results suggested the possibility that these 30 highly fluctuating residues along with some hydrophobic and dimer interface residues in surface exhibit dynamic nature by the deletion. A hypothesis is that the fluctuation of these unstable residues might induce the formation of locally unfolded states, which can initiate aggregation or multimerization of s-DL4.

## Discussion

In this study, it has been shown that the deletional protein engineering approach can be more efficiently performed by using a more stabilized protein structure. This was demonstrated by comparing two GFP sequences with different stabilities, n-GFP and s-GFP. In addition, the deletion mutagenesis of s-GFP with high stability also enabled us to generate deletion mutants such as s-N14, s-C225 and s-DL4, which cannot be obtained from normal GFP fold. s-N14 is the 2–14 residues deleted mutant of s-GFP without the small turn, one helix and a part of first β-strand in N-terminus of GFP. To our knowledge, s-N14 is the first GFP mutant showing fluorescent activity without the amino acid residues forming the first turn and helix. The deletion of 8 residues in the GFP N-terminus has been known to abolish the GFP activity completely so far [6]. The C-terminus deletion mutant of 225–238, s-C225, is also likely to be the first deletion mutant without a part of the last β-strand and complete tail sequence but showing fluorescence activity. In the previous study, the maximum deletion for C-terminus was the deletion of 229–238 residues while retaining its functional activity [6]. In the study, internal loops deletions were made but none of the loop deleted variants were found to be fluorescent, whereas deletion mutant of internal loop (191GPVLLP196), s-DL4, showed fluorescent activity in our study. Various types of split GFPs and truncated circularly permuted GFPs were reported to fluoresce [42], [43]. However, in those studies, only excisions were made to prepare two fragments and the two fragments were reassembled to form complete β-barrel structure, whereas the variants were functionally active after true deletions in our study.

Though the tendency of a protein to form aggregate under physiological conditions is unpredictable yet, aggregation can happen through thermal fluctuations occurring under physiological conditions [44], [45]. For example, an enzyme acylphosphatase from *Sulfolobus solfataricus* have been studied extensively and found to form active native-like aggregates through partially unfolded protein molecules [46], [47], [48]. These results support our hypothesis for the aggregation of active s-DL4 variant that the dynamic fluctuations of some hydrophobic residues in exposed positions or dimer interface residues in GFP can induce such aggregation. However, detailed structural characterization of the s-DL4 by NMR spectroscopy or X-ray diffraction may be needed to give the exact reason for the aggregation of the s-DL4.

Our study focused on demonstrating that deletion mutagenesis can be more efficiently performed using stable fold by performing terminal deletions and loop deletions. We performed relatively systematic deletion studies for N-terminal and C-terminal residues, and found the important residues in respective terminals that are essential for retaining the native structure of stable GFP. On the other hand, the loop deletion mutagenesis strategy was not so systematic and just adapted from the previous work to compare the tolerance of deletion mutation between s-GFP and n-GFP. Further systematic deletion studies for the loop regions are needed to understand more exactly how such mutations have influence on the protein structure. Different type of deletion studies such as deletions in β-strands are also expected to provide chances to generate different interesting variants.

Although the engineering of physical properties such as protein size and protein aggregation were shown in our study, the approach using stabilized protein may also facilitate the engineering of catalytic proteins through deletion mutagenesis. For example, it has been reported that the aminopeptidase PepC was engineered by deleting four amino acids in C-terminal, which resulted in a new enzyme with altered property [49]. This demonstrated that deletion mutagenesis can be used for changing catalytic activities of protein. Therefore, in the case that the deletions for catalytic activity changes are related with protein structure formation, the improvement of protein stability may give us more chance to achieve active protein with altered catalytic properties.

In conclusion, this study demonstrated that the deletional protein engineering approach can be more efficiently performed by using a more stabilized protein structure. This indicates that the concept of protein stability-evolvability relationship can be applied to overcome the problem of deletional protein engineering. Our study also demonstrates that the deletion mutagenesis can generate a sequence-structure space different from the space derived by traditional substitution mutagenesis, which allows us to obtain the variants that cannot be obtained from the usual substitution mutagenesis. For instance, the functionally active insoluble s-DL4 looks like a unique GFP variant that has not been obtained from the tremendous previous GFP engineering studies mainly based on substitution mutagenesis. It is expected that our study can be helpful to facilitate the use of deletion method in protein engineering.

## Supporting Information

Figure S1 Relative whole cell fluorescence activity of combinatorial deletion. Relative whole cell fluorescence (in arbitrary units) of combined deletion of N-terminal, C-terminal and internal loops (191–196) of s-GFP. The fluorescence of *E.coli* BL21 (DE3) expressing deletion variants was measured after the reached O.D_600_ of 0.6 at 37°C was induced with 1 mM IPTG for 5 hrs. The relative fluorescence (in arbitrary units) is defined as the whole cell fluorescence compared with fluorescence of cells expressing s-GFP. All the fluorescence values were normalized by the O.D_600 nm_ of expressed cells.
(TIF)

Figure S2 SDS-PAGE analysis of expressed protein. Expression profiles of designed s-GFP and n-GFP variants. Solid head arrow mark shows the expressed proteins in all images. T-Total cell protein, S-Soluble fraction, I-Insoluble fraction. Expression and solubility level of a) Internal loop deletions b) N-terminal deletions c) C-terminal deletions and d) Combined deletions of s-GFP. SDS-PAGE analysis and solubility level of e) N-terminal deletions f) combined deletions and internal loop deletion (n-DL4) of n-GFP variants.
(TIF)

Supporting Information S1 (DOC)

Table S1 Estimated Secondary structure content recovered through DichroWeb server.
(TIF)

Table S2 Lost and new interactions of N-terminal deletion mutants (s-N11, s-N14).
(TIF)

Table S3 Change in interactions of C-terminal deletion mutants (s-C225, s-C224).
(TIF)

Table S4 Lost and new electrostatic interactions in both terminals combined deletion mutants (s-N11C227).
(TIF)

Table S5 Lost Hydrogen bonds and salt bridges of internal loop deletions s-DL1 and s-DL2.
(TIF)

Table S6 Fluctuating amino acids in s-DL4.
(TIF)

## References

[1] DePristo MA, Weinreich DM, Hartl DL (2005) Missense meanderings in sequence space: a biophysical view of protein evolution. Nat Rev Genet 6: 678–687. 16074985 10.1038/nrg1672

[2] Camps M, Herman A, Loh E, Loeb LA (2007) Genetic constraints on protein evolution. Crit Rev Biochem Mol Biol 42: 313–326. 17917869 10.1080/10409230701597642 PMC3825456

[3] Povolotskaya IS, Kondrashov FA (2010) Sequence space and the ongoing expansion of the protein universe. Nature 465: 922–926. 20485343 10.1038/nature09105

[4] Shortle D, Sondek J (1995) The emerging role of insertions and deletions in protein engineering. Curr Opin Biotechnol 6: 387–393. 7579648 10.1016/0958-1669(95)80067-0

[5] Dopf J, Horiagon TM (1996) Deletion mapping of the Aequorea victoria green fluorescent protein. Gene 173: 39–44. 8707054 10.1016/0378-1119(95)00692-3

[6] Li X, Zhang G, Ngo N, Zhao X, Kain SR, et al (1997) Deletions of the Aequorea victoria green fluorescent protein define the minimal domain required for fluorescence. J Biol Chem 272: 28545–28549. 9353317 10.1074/jbc.272.45.28545

[7] Santhoshkumar P, Murugesan R, Sharma KK (2009) Deletion of ^54^FLRAPSWF^61^ residues decreases the oligomeric size and enhances the chaperone function of αB-crystallin. Biochemistry 48: 5066–5073. 19388699 10.1021/bi900085v PMC3997080

[8] Gong R, Wang Y, Feng Y, Zhao Q, Dimitrov DS (2011) Shortened engineered human antibody CH2 domains. J Biol Chem 286: 27288–27293. 21669873 10.1074/jbc.M111.254219 PMC3149322

[9] Vamvaca K, Lansbury Jr PT, Stefanis L (2011) N-terminal deletion does not affect α-synuclein membrane binding, self-association and toxicity in human neuroblastoma cells, unlike yeast. J Neurochem 119: 389–397. 21848810 10.1111/j.1471-4159.2011.07431.x PMC3432859

[10] Haglund E, Danielsson J, Kadhirvel S, Lindberg MO, Logan DT, et al (2012) Trimming down a protein structure to its bare foldons. J Biol Chem 287: 2731–2738. 22117065 10.1074/jbc.M111.312447 PMC3268430

[11] Liu L, Zhang G, Zhang Z, Wang S, Chen H (2011) Terminal amino acids disturb xylanase thermostability and activity. J Biol Chem 286: 44710–44715. 22072708 10.1074/jbc.M111.269753 PMC3247970

[12] Trevino RJ, Tsalkova T, Kramer G, Hardesty B, Chirgwin JM, et al (1998) Truncations at the NH_2_-terminus of rhodanese destabilize the enzyme and decrease its heterologous expression. J Biol Chem 273: 27841–27847. 9774394 10.1074/jbc.273.43.27841

[13] Trevino RJ, Gliubich F, Berni R, Cianci M, Chirgwin JM, et al (1999) NH_2_-terminal sequence truncation decreases the stability of bovine rhodanese, minimally perturbs its crystal structure, and enhances interaction with GroEL under native conditions. J Biol Chem 274: 13938–13947. 10318804 10.1074/jbc.274.20.13938

[14] Putnam-Evans C, Wu J, Bricker TM (1996) Site-directed mutagenesis of the CP 47 protein of photosystem II: alteration of conserved charged residues which lie within lethal deletions of the large extrinsic loop E. Plant Mol Biol. 32: 1191–1195. 10.1007/BF00041405 9002620

[15] Bloom JD, Labthavikul ST, Otey CR, Arnold FH (2006) Protein stability promotes evolvability. Proc Natl Acad Sci U S A 103: 5869–5874. 16581913 10.1073/pnas.0510098103 PMC1458665

[16] Tokuriki N, Tawfik DS (2009) Stability effects of mutations and protein evolvability. Curr Opin Struct Biol 19: 596–604. 19765975 10.1016/j.sbi.2009.08.003

[17] Caetano-Anollés G, Mittenthal J (2010) Exploring the interplay of stability and function in protein evolution. BioEssays 32: 655–658. 20658703 10.1002/bies.201000038

[18] Bloom JD, Wilke CO, Arnold FH, Adami C (2004) Stability and the evolvability of function in a model protein. Biophys J 86: 2758–2764. 15111394 10.1016/S0006-3495(04)74329-5 PMC1304146

[19] Müller MM, Kries H, Csuhai E, Kast P, Hilvert D (2010) Design, selection, and characterization of a split chorismate mutase. Protein Sci 19: 1000–1010. 20306491 10.1002/pro.377 PMC2868242

[20] Leconte AM, Patel MP, Sass LE, McInerney P, Jarosz M, et al (2010) Directed evolution of DNA polymerases for next-generation sequencing. Angew Chem Int Ed Engl 49: 5921–5924. 20629059 10.1002/anie.201001607 PMC3043640

[21] Li Y, Drummond DA, Sawayama AM, Snow CD, Bloom JD, et al (2007) A diverse family of thermostable cytochrome P450s created by recombination of stabilizing fragments. Nat Biotechnol 25: 1051–1056. 17721510 10.1038/nbt1333

[22] Nagasundarapandian S, Merkel L, Budisa N, Govindan R, Ayyadurai N, et al (2010) Engineering protein sequence composition for folding robustness renders efficient noncanonical amino acid incorporations. ChemBioChem 11: 2521–2524. 21064080 10.1002/cbic.201000380

[23] Ayyadurai N, Saravanan Prabhu N, Deepankumar K, Lee S-G, Jeong H-H, et al (2011) Development of a selective, sensitive, and reversible biosensor by the genetic incorporation of a metal-binding site into green fluorescent protein. Angew Chem Int Ed Engl 50: 6534–6537. 21656613 10.1002/anie.201008289

[24] Ayyadurai N, Prabhu NS, Deepankumar K, Jang YJ, Chitrapriya N, et al (2011) Bioconjugation of l-3,4-dihydroxyphenylalanine containing protein with a polysaccharide. Bioconjug Chem 22: 551–555. 21375304 10.1021/bc2000066

[25] Ayyadurai N, Neelamegam R, Nagasundarapandian S, Edwardraja S, Park H, et al (2009) Importance of expression system in the production of unnatural recombinant proteins in Escherichia coli. Biotechnol Bioprocess Eng 14: 257–265.

[26] Higuchi R, Krummel B, Saiki R (1988) A general method of in vitro preparation and specific mutagenesis of DNA fragments: study of protein and DNA interactions. Nucleic Acids Res 16: 7351–7367. 3045756 10.1093/nar/16.15.7351 PMC338413

[27] Pedelacq J-D, Cabantous S, Tran T, Terwilliger TC, Waldo GS (2006) Engineering and characterization of a superfolder green fluorescent protein. Nat Biotechnol 24: 79–88. 16369541 10.1038/nbt1172

[28] Whitmore L, Wallace BA (2008) Protein secondary structure analyses from circular dichroism spectroscopy: Methods and reference databases. Biopolymers 89: 392–400. 17896349 10.1002/bip.20853

[29] Whitmore L, Wallace BA (2004) DICHROWEB, an online server for protein secondary structure analyses from circular dichroism spectroscopic data. Nucleic Acids Res 32: W668–W673. 15215473 10.1093/nar/gkh371 PMC441509

[30] Barlow DJ, Thornton JM (1983) Ion-pairs in proteins. J Mol Biol 168: 867–885. 6887253 10.1016/s0022-2836(83)80079-5

[31] McDonald IK, Thornton JM (1994) Satisfying hydrogen bonding potential in proteins. J Mol Biol 238: 777–793. 8182748 10.1006/jmbi.1994.1334

[32] Yang F, Moss LG, Phillips GN (1996) The molecular structure of green fluorescent protein. Nat Biotechnol 14: 1246–1251. 9631087 10.1038/nbt1096-1246

[33] Hess B, Kutzner C, van der Spoel D, Lindahl E (2008) GROMACS 4: Algorithms for highly efficient, load-balanced, and scalable molecular simulation. J Chem Theory Comput 4: 435–447. 26620784 10.1021/ct700301q

[34] Cormack BP, Valdivia RH, Falkow S (1996) FACS-optimized mutants of the green fluorescent protein (GFP). Gene 173: 33–38. 8707053 10.1016/0378-1119(95)00685-0

[35] Bulter T, Lee S-G, Wong WW, Fung E, Connor MR, et al (2004) Design of artificial cell–cell communication using gene and metabolic networks. Proc Natl Acad Sci U S A 101: 2299–2304. 14983004 10.1073/pnas.0306484101 PMC356945

[36] Fung E, Wong WW, Suen JK, Bulter T, Lee S-g, et al (2005) A synthetic gene-metabolic oscillator. Nature 435: 118–122. 15875027 10.1038/nature03508

[37] Ormö M, Cubitt AB, Kallio K, Gross LA, Tsien RY, et al (1996) Crystal structure of the Aequorea victoria green fluorescent protein. Science 273: 1392–1395. 8703075 10.1126/science.273.5280.1392

[38] Sample V, Newman RH, Zhang J (2009) The structure and function of fluorescent proteins. Chem Soc Rev 38: 2852–2864. 19771332 10.1039/b913033k

[39] Tsien RY (1998) The green fluorescent protein. Annu Rev Biochem 67: 509–544. 9759496 10.1146/annurev.biochem.67.1.509

[40] Ehrig T, O’Kane DJ, Prendergast FG (1995) Green fluorescent protein mutants with altered fluorescence excitation spectra. FEBS Letters 367: 163–166. 7796912 10.1016/0014-5793(95)00557-p

[41] Peternel S, Grdadolnik J, Gaberc-Porekar V, Komel R (2008) Engineering inclusion bodies for non denaturing extraction of functional proteins. Microb Cell Fact 7: 34. 19046444 10.1186/1475-2859-7-34 PMC2630956

[42] Huang Y-M, Nayak S, Bystroff C (2011) Quantitative in vivo solubility and reconstitution of truncated circular permutants of green fluorescent protein. Protein Sci 20: 1775–1780. 21910151 10.1002/pro.735 PMC3267941

[43] Kent KP, Oltrogge LM, Boxer SG (2009) Synthetic control of green fluorescent protein. J Am Chem Soc 131: 15988–15989. 19839621 10.1021/ja906303f PMC2783612

[44] Chiti F, Dobson CM (2009) Amyloid formation by globular proteins under native conditions. Nat Chem Biol 5: 15–22. 19088715 10.1038/nchembio.131

[45] Ventura S, Villaverde A (2006) Protein quality in bacterial inclusion bodies. Trends Biotechnol 24: 179–185. 16503059 10.1016/j.tibtech.2006.02.007

[46] Bemporad F, Vannocci T, Varela L, Azuaga AI, Chiti F (2008) A model for the aggregation of the acylphosphatase from Sulfolobus solfataricus in its native-like state. Biochim Biophys Acta 1784: 1986–1996. 18832052 10.1016/j.bbapap.2008.08.021

[47] Pagano K, Bemporad F, Fogolari F, Esposito G, Viglino P, et al (2010) Structural and dynamics characteristics of acylphosphatase from Sulfolobus solfataricus in the monomeric state and in the Initial native-like aggregates. J Biol Chem 285: 14689–14700. 20223823 10.1074/jbc.M109.082156 PMC2863212

[48] Plakoutsi G, Bemporad F, Monti M, Pagnozzi D, Pucci P, et al (2006) Exploring the mechanism of formation of native-like and precursor amyloid oligomers for the native acylphosphatase from Sulfolobus solfataricus. Structure 14: 993–1001. 16765892 10.1016/j.str.2006.03.014

[49] Mata L, Gripon J-C, Mistou M-Y (1999) Deletion of the four C-terminal residues of PepC converts an aminopeptidase into an oligopeptidase. Protein Engineering 12: 681–686. 10469829 10.1093/protein/12.8.681

