# Supplementary figures and images for: Deletional Protein Engineering Based on Stable Fold
Source: PLoS One. 2012 Dec 11;7(12):e51510. doi: 10.1371/journal.pone.0051510 (PMC3519881; doi:10.1371/journal.pone.0051510)

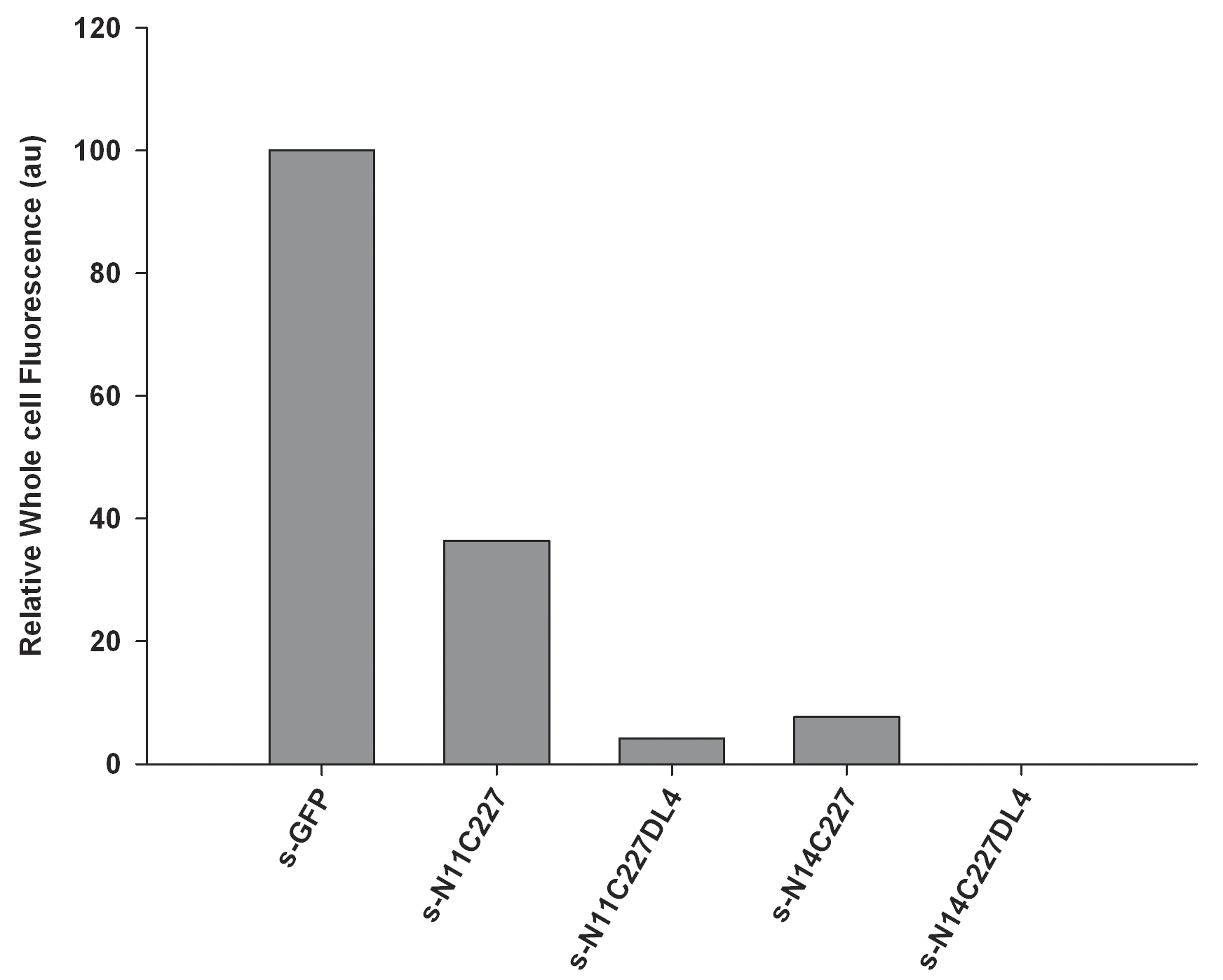

Supplement: Figure S1 — Relative whole cell fluorescence activity of combinatorial deletion. Relative whole cell fluorescence (in arbitrary units) of combined deletion of N-terminal, C-terminal and internal loops (191–196) of s-GFP. The fluorescence of E.coli BL21 (DE3) expressing deletion variants was measured after the reached O.D600 of 0.6 at 37°C was induced with 1 mM IPTG for 5 hrs. The relative fluorescence (in arbitrary units) is defined as the whole cell fluorescence compared with fluorescence of cells expressing s-GFP. All the fluorescence values were normalized by the O.D600 nm of expressed cells. (TIF) [file pone.0051510.s001.tif]

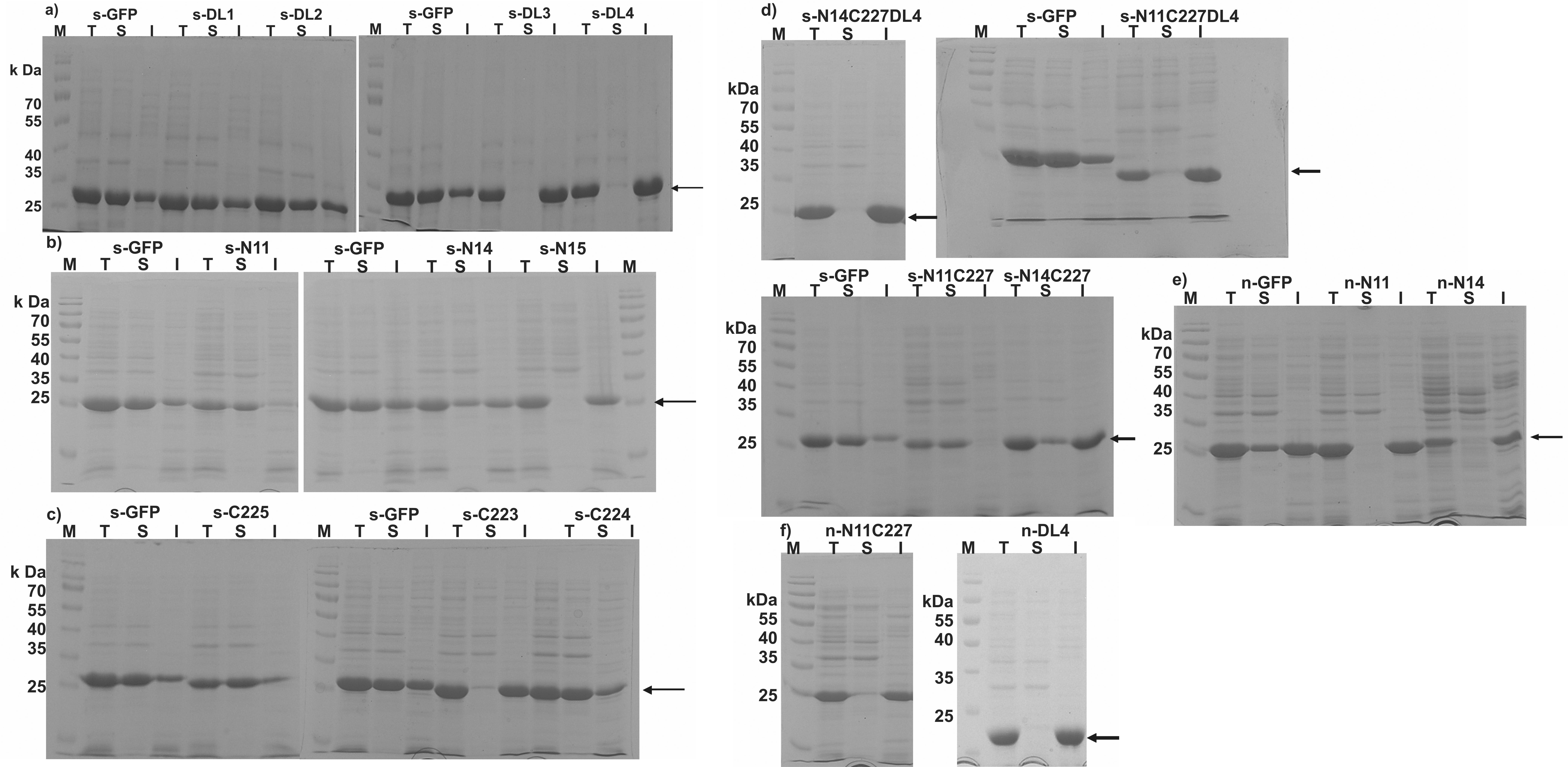

Supplement: Figure S2 — SDS-PAGE analysis of expressed protein. Expression profiles of designed s-GFP and n-GFP variants. Solid head arrow mark shows the expressed proteins in all images. T-Total cell protein, S-Soluble fraction, I-Insoluble fraction. Expression and solubility level of a) Internal loop deletions b) N-terminal deletions c) C-terminal deletions and d) Combined deletions of s-GFP. SDS-PAGE analysis and solubility level of e) N-terminal deletions f) combined deletions and internal loop deletion (n-DL4) of n-GFP variants. (TIF) [file pone.0051510.s002.tif]

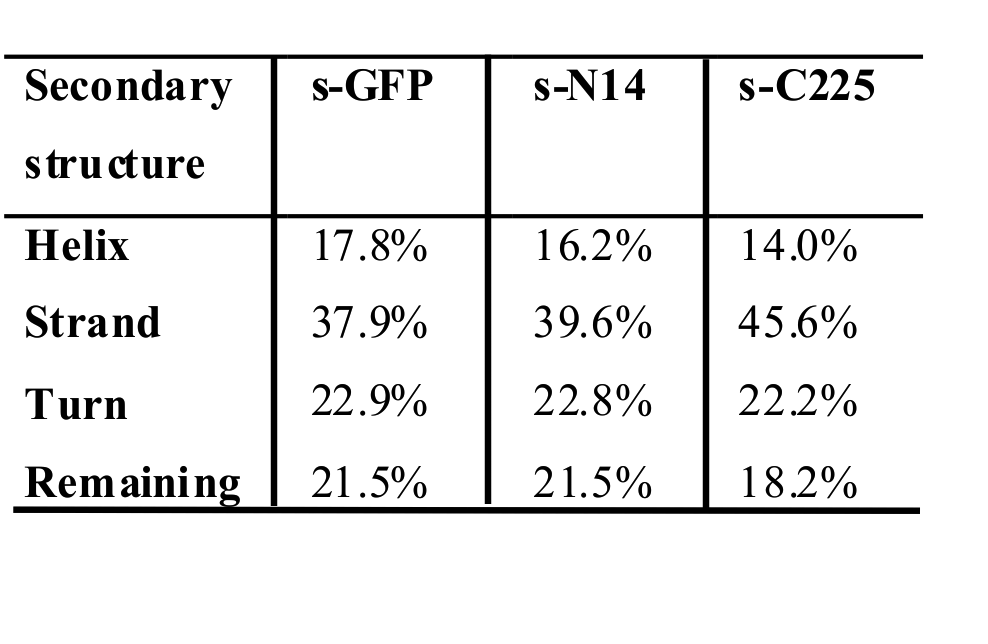

Supplement: Table S1 — Estimated Secondary structure content recovered through DichroWeb server. (TIF) [file pone.0051510.s004.tif]

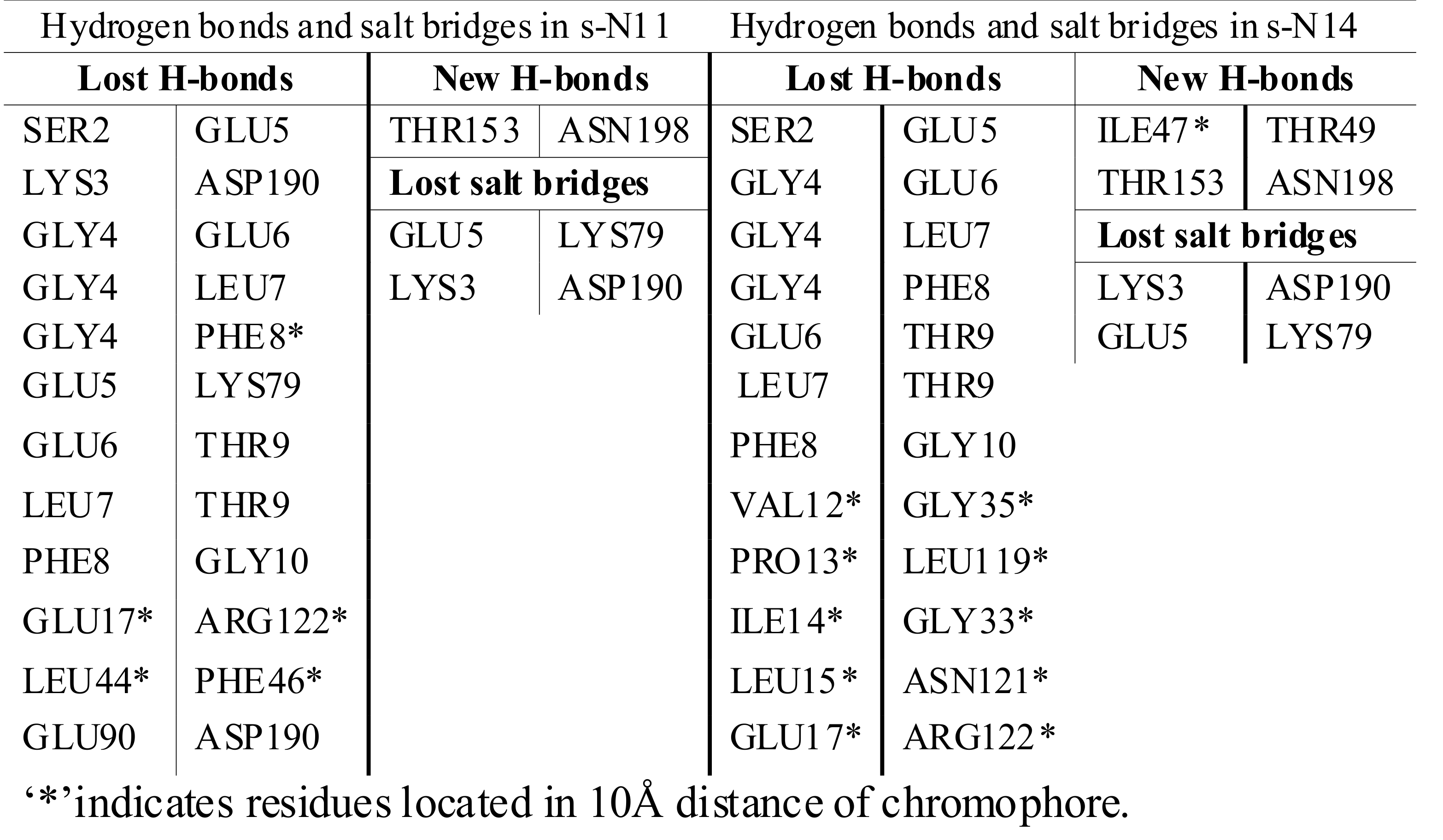

Supplement: Table S2 — Lost and new interactions of N-terminal deletion mutants (s-N11, s-N14). (TIF) [file pone.0051510.s005.tif]

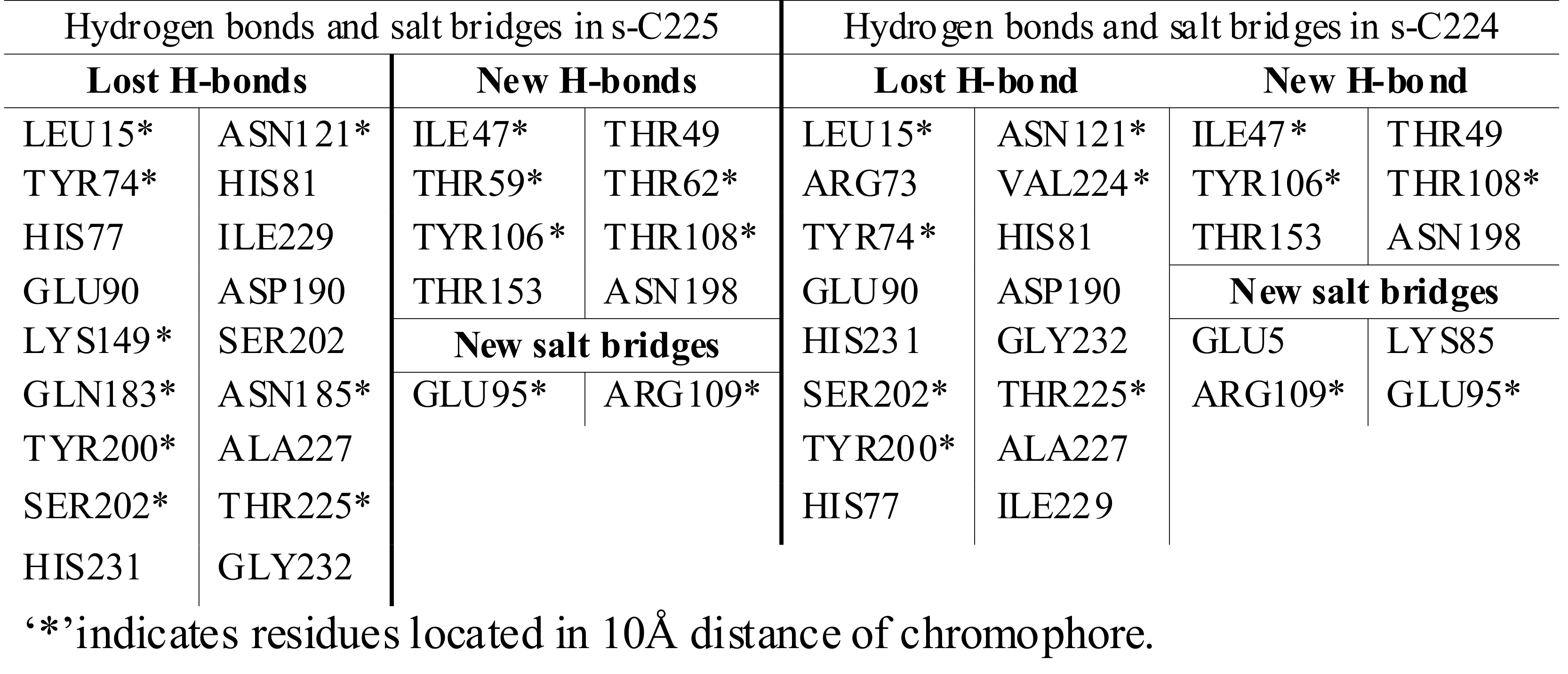

Supplement: Table S3 — Change in interactions of C-terminal deletion mutants (s-C225, s-C224). (TIF) [file pone.0051510.s006.tif]

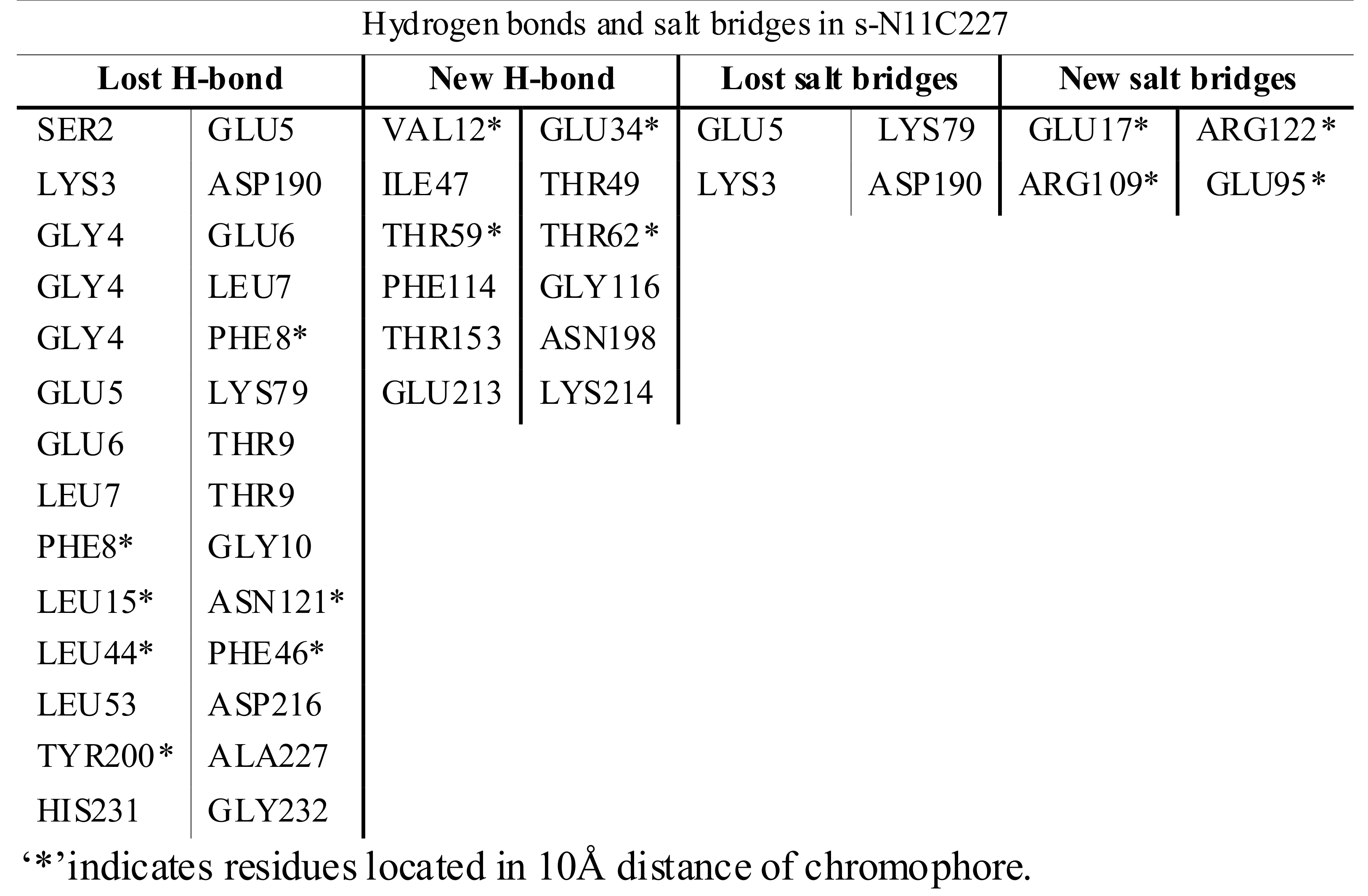

Supplement: Table S4 — Lost and new electrostatic interactions in both terminals combined deletion mutants (s-N11C227). (TIF) [file pone.0051510.s007.tif]

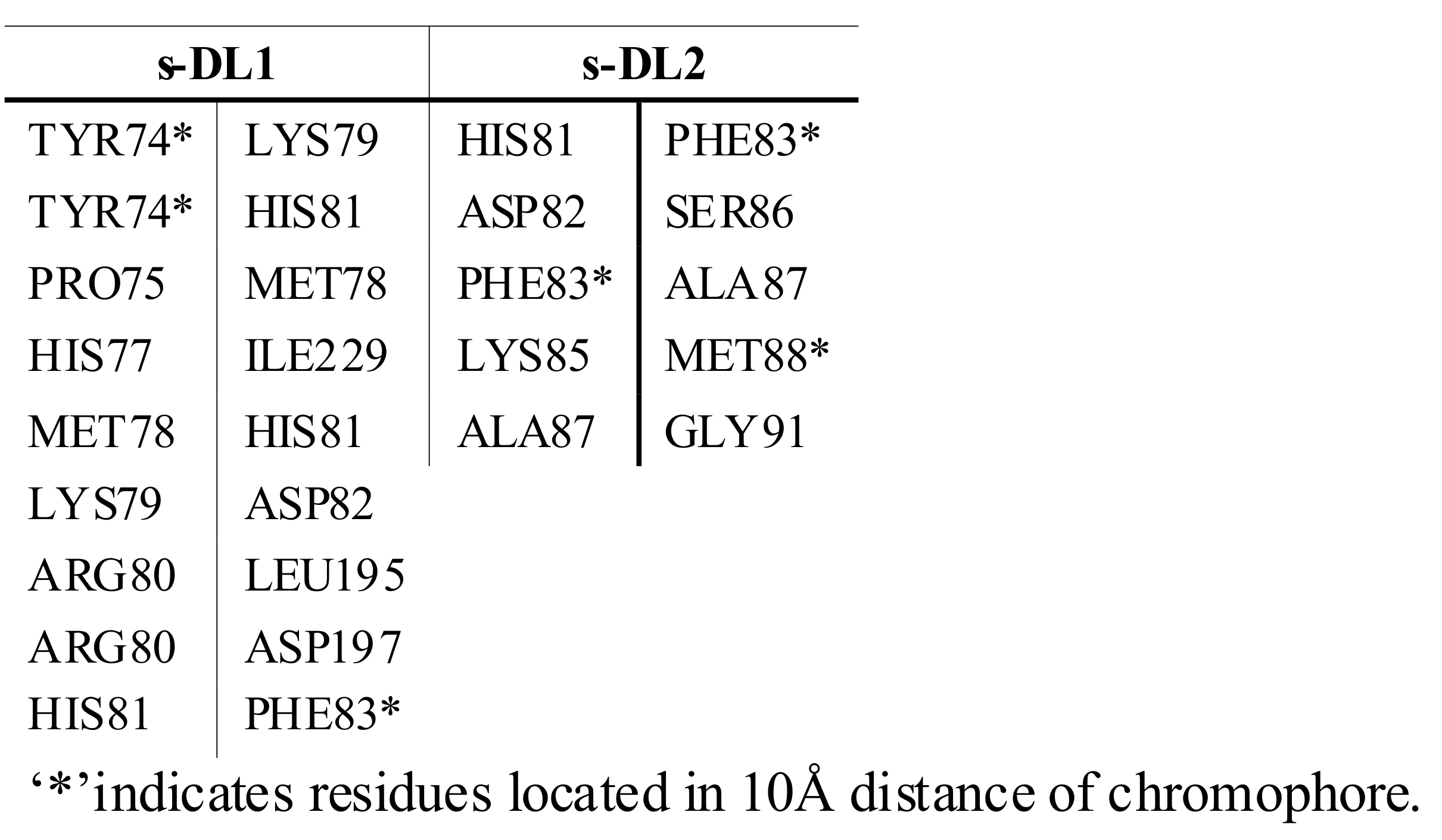

Supplement: Table S5 — Lost Hydrogen bonds and salt bridges of internal loop deletions s-DL1 and s-DL2. (TIF) [file pone.0051510.s008.tif]

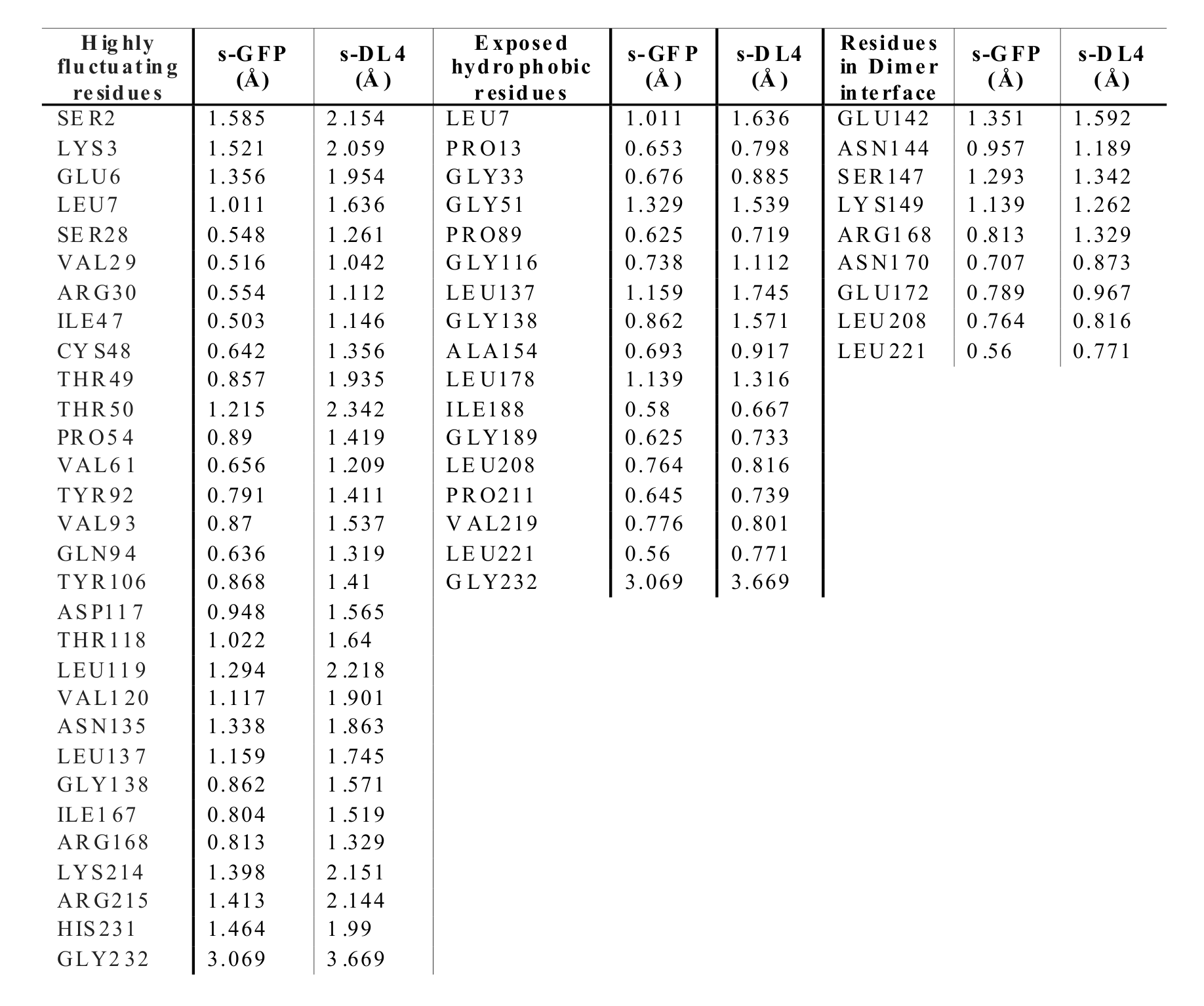

Supplement: Table S6 — Fluctuating amino acids in s-DL4. (TIF) [file pone.0051510.s009.tif]
