# Supplementary material for: Deletional Protein Engineering Based on Stable Fold
Source: PLoS One. 2012 Dec 11;7(12):e51510. doi: 10.1371/journal.pone.0051510 (PMC3519881; doi:10.1371/journal.pone.0051510)
Supplement: Supporting Information S1 — (DOC) [file pone.0051510.s003.doc]

**Supplementary Information S1**

**Primers used in the preparation of deletion variants**

**<Primers for N- and C-terminal deletions of s-GFP>**

| **N-terminal deletion:** | |
| --- | --- |
| s-N11-FOR | 5’GATCATATGGTGCCGATTCTGGTGGAACTG 3’ |
| s-N14-FOR | 5’ GATCATATGCTGGTGGAACTGGATGGCG 3’ |
| s-N15-FOR | 5’GATCATATGGTGGAACTGGATGGCGATGTG 3’ |
| s-GFP-REV | 5’GATCTCGAGTTATTAATGGTGATGGTGATGGTG 3’ |
| **C-terminal deletion:** | |
| s-GFP-FOR | 5’GATCATATGCACCATCACCATCACCATAGCAAAGGCGAAGAACTGTTTAC ’3 |
| s-C223-REV | 5’ GTACTCGAGTTATTATTCCAGCAGCACCATGTGAT 3’ |
| s-C224-REV | 5’ GTACTCGAGTTATTAAAATTCCAGCAGCACCATGTG 3’ |
| s-C225-REV | 5’ GTACTCGAGTTATTACACAAATTCCAGCAGCACCATG 3’ |
| s-C226-REV | 5’ GTACTCGAGTTATTAGGTCACAAATTCCAGCAGCA 3’ |
| s-C227-REV | 5’GTACTCGAGTTATTAATGGTGATGGTGATGGTGCGCGGTCACAAATTCCAG 3’ |

<**Primers for N- and C-terminal deletions of n-GFP>**

| **N-terminal deletions:** | |
| --- | --- |
| n-GFP-FOR | 5’ GAT CATATGCAGTCTAAAGGAGAAGAAC 3’ |
| s-GFP-REV | 5’GATCTCGAGTTATTAATGGTGATGGTGATGGTG 3’ |
| n-N11-FOR | 5’ GAT CATATG GTCCCAATTCTTGTTGAATTAGATGG 3’ |
| n-N14-FOR | 5’ GAT CATATG CTTGTTGAATTAGATGGTGATGTTAATG 3’ |
| **C-terminal deletions:** | |
| n-GFP-FOR | 5’GATCATATGCACCATCACCATCACCATTCTAAAGGAGAAGAACTTTTCACTG 3' |
| n-C225-REV | 5'GTACTCGAGTTATTATACAAACTCAAGAAGGACCATGTG 3' |
| n-C227-REV | 5’GTACTCGAGTTATTAATGGTGATGGTGATGGTGAGCTGTTACAAACTCAAGAAGGAC 3’ |

**<Primers for internal loop deletions of s-GFP>**

| **s-DL1** | |
| --- | --- |
| s-GFP-FOR | 5’GATCATATGCAGAGCAAAGGCG 3’ |
| GFPhs1del76-REV | 5’CGGATAACGTGCAAAGCACTGC 3’ |
| GFPhs1del81-FOR | 5’CGTTATCCGGATTTCTTTAAAAGCGCGATGCC 3’ |
| s-GFP-REV | 5’GATCTCGAGTTATTAATGGTGATGGTGATGGTG 3’ |
| **s-DL2** | |
| s-GFP-FOR | 5’GATCATATGCAGAGCAAAGGCG 3’ |
| GFPhs1del83-REV | 5’ ATCATGACGTTTCATGTGATCCG 3’ |
| GFPhs1del88-FOR | 5’CGTCATGATCCGGAAGGCTATGTGCAGG 3’ |
| s-GFP-REV | 5’GATCTCGAGTTATTAATGGTGATGGTGATGGTG3’ |
| **s-DL3** | |
| s-GFP-FOR | 5’GATCATATGCAGAGCAAAGGCG 3’ |
| GFPhs1del132-REV | 5’TTTAAAATCAATGCCTTTCAGTTCAATACGG’3 |
| GFPhs1del139-FOR | 5’ GATTTTAAAAAACTGGAATATAACTTTAACAGCC 3’ |
| s-GFP-REV | 5’GATCTCGAGTTATTAATGGTGATGGTGATGGTG’3 |
| **s-DL4** | |
| s-GFP-FOR | 5’GATCATATGCAGAGCAAAGGCG 3’ |
| GFPhs1del191-REV | 5’ATCGCCAATCGGGGTGTTCTG 3’ |
| GFPhs1del197-FOR | 5’GATTGGCGATGATAACCATTATCTGAGCACCCAGAG 3’ |
| s-GFP-REV | 5’GATCTCGAGTTATTAATGGTGATGGTGATGGTG 3’ |

The gene for n-DL4 gene alone was synthesized commercially from Genescript Corporation (New Jersey, USA).

**Gene sequence of s-GFP and n-GFP**

>s-GFP

CATATGCAGAGCAAAGGCGAAGAACTGTTTACCGGCGTGGTGCCGATTCTGGTGGAACTG

GATGGCGATGTGAACGGCCATAAATTTAGCGTGCGTGGCGAAGGCGAAGGCGATGCGACC

AACGGCAAACTGACCCTGAAATTTATTTGCACCACCGGTAAACTGCCGGTGCCGTGGCCG

ACCCTGGTGACCACCCTGGGTTATGGTGTGCAGTGCTTTGCACGTTATCCGGATCACATG

AAACGTCATGATTTCTTTAAAAGCGCGATGCCGGAAGGCTATGTGCAGGAACGTACCATT

AGCTTTAAAGATGATGGCACCTATAAAACCCGTGCGGAAGTGAAATTTGAAGGCGATACC

CTGGTGAACCGTATTGAACTGAAAGGCATTGATTTTAAAGAAGATGGCAACATTCTGGGC

CATAAACTGGAATATAACTTTAACAGCCATAAAGTGTATATTACCGCGGATAAACAGAAA

AACGGCATTAAAGCGAACTTTAAAATTCGTCATAACGTGGAAGATGGCAGCGTGCAGCTG

GCGGATCATTATCAGCAGAACACCCCGATTGGCGATGGCCCGGTGCTGCTGCCGGATAAC

CATTATCTGAGCACCCAGAGCGTGCTGCTGAAAGATCCGAACGAAAAACGTGATCACATG

GTGCTGCTGGAATTTGTGACCGCGGCGGGCATTACCCACGGCATGGATGAACTGTATAAA

CACCATCACCATCACCATTAATAACTCGAG

>n-GFP

CATATGCAGTCTAAAGGAGAAGAACTTTTCACTGGAGTTGTCCCAATTCTTGTTGAATTA

GATGGTGATGTTAATGGGCACAAATTTTCTGTCAGTGGAGAGGGTGAAGGTGATGCAACA

TACGGAAAACTTACCCTTAAATTTATTTGCACTACTGGAAAACTACCTGTTCCGTGGCCA

ACACTTGTCACTACTTTCGGTTATGGTGTTCAATGCTTTGCGAGATACCCAGATCACATG

AAACAGCATGACTTTTTCAAGAGTGCCATGCCCGAAGGTTACGTACAGGAAAGAACTATA

TTTTTCAAAGATGACGGGAACTACAAGACACGTGCTGAAGTCAAGTTTGAAGGTGATACC

CTTGTTAATAGAATCGAGTTAAAAGGTATTGATTTTAAAGAAGATGGAAACATTCTTGGA

CACAAATTGGAATACAACTATAACTCACACAATGTATACATCATGGCAGACAAACAAAAG

AATGGAATCAAAGTTAACTTCAAAATTAGACACAACATTGAAGATGGAAGCGTTCAACTA

GCAGACCATTATCAACAAAATACTCCAATTGGCGATGGCCCTGTCCTTTTACCAGACAAC

CATTACCTGTCCACACAATCTGCCCTTTCGAAAGATCCCAACGAAAAGAGAGACCACATG

GTCCTTCTTGAGTTTGTAACAGCTGCTGGGATTACACATGGCATGGATGAACTATACAAA

CACCATCACCATCACCATTAATAACTCGAG
